# Safety, tolerability, and immunogenicity of a DNA-based vaccine (INO-4700) against Middle East respiratory syndrome coronavirus: phase 2a study in healthy volunteers

**DOI:** 10.3389/fimmu.2025.1662923

**Published:** 2025-11-14

**Authors:** Joseph T. Agnes, Sarah A. Marcus, Sahem S. Al-Ghraibeh, Suleimman Ahmad Al-Sweedan, Josphat Kosgei, Bernhards Ogutu, ShuPing Yang, Kathleen A. Walker, Bonaventure Orizu, Kate E. Broderick, Jean Boyer, Stephanie Ramos, Matthew P. Morrow, Kimberly Kraynyak, Albert J. Sylvester, Elisabeth Gillespie, David Liebowitz, Laurent M. Humeau

**Affiliations:** 1 Inovio Pharmaceuticals, Inc., Plymouth Meeting, PA, United States; 2 Department of Internal Medicine, Faculty of Medicine, Yarmouk University, Irbid, Jordan; 3 Department of Pediatric Hematology/Oncology/BMT, King Abdullah University Hospital, Faculty of Medicine, Jordan University of Science & Technology, Ar Ramtha, Irbid, Jordan; 4 Kenya Medical Research Institute/Walter Reed Project Clinical Research Center, Kericho Clinical Research Site, Kericho, Kenya; 5 Kenya Medical Research Institute (KEMRI), Nairobi, Kenya

**Keywords:** DNA medicine, Middle east respiratory syndrome (MERS), MERS, safety, immunogenicity, electroporation (EP), vaccine

## Abstract

**Background:**

Middle East respiratory syndrome coronavirus (MERS-CoV) poses an ongoing public health risk with a 36% case-fatality rate and no licensed vaccines. This Phase 2a, randomized, blinded, placebo-controlled, multi-center trial (MERS-201; NCT04588428) evaluated the safety, tolerability, and immunogenicity of INO-4700, a DNA vaccine against the MERS-CoV spike glycoprotein, in healthy adult volunteers.

**Methods:**

Participants received INO-4700 or placebo intradermally followed by electroporation upon enrollment into any one of five active treatment groups, resulting from three-dose levels (0.6 mg, 1 mg, and 2 mg total) during each of two dosing days or four placebo groups. Doses were administered as 1 or 2 concurrent injections to achieve the total dose level at Week 0 and at either Week 4 or 8. Safety endpoints included incidence of treatment-emergent adverse events (TEAEs), their toxicity grading scale, seriousness, and relationship to study treatment and AEs of special interest (AESI). Immunogenicity endpoints included evaluation of humoral and cellular immune responses, assessed pre-dose (Screening and/or Week 0) and at Weeks 6 and 10.

**Results:**

One hundred and ninety-two participants were randomized across the nine study groups and followed up between June 2021 and January 2023. Treatment with INO-4700 was well-tolerated and had a favorable safety profile with low incidence of TEAEs, which were overall similar between INO-4700 and placebo groups, with most of the TEAEs assessed as Grade 1 or Grade 2, non-serious, and unrelated to treatment. Group E, the highest INO-4700 dose tested (2 mg total), showed greater immune responses compared to other groups, with significantly elevated MERS-CoV receptor-binding domain (RBD) and spike-binding IgG levels, and seroreactivity at Week 10 peaking at 42% and 32%, respectively. Spike-specific T cell responses further contributed to INO-4700 immunogenicity, ranging from 29% in Group C to 50% in Group E.

**Conclusions:**

DNA vaccine INO-4700 was well-tolerated in healthy adults across all groups after each dose was administered and elicited humoral and cellular immune responses. These results warrant further evaluation of INO-4700 as a candidate vaccine for MERS-CoV outbreak preparedness and prevention.

**Clinical Trial Registration:**

https://clinicaltrials.gov, **identifier NCT04588428**.

## Introduction

The Middle East respiratory syndrome coronavirus (MERS-CoV) has persisted as a significant global health concern since its initial emergence (1). First identified in Saudi Arabia in 2012 – seven years before the emergence of severe acute respiratory syndrome coronavirus 2 (SARS-CoV-2) in China – MERS-CoV drew global attention due to its epidemic potential, high mortality rate and zoonotic origin (1–3). The unprecedented global mobilization in response to the SARS-CoV-2 pandemic has also accelerated the accumulation of data relevant to MERS-CoV research and treatment strategies (1). Transmission of the virus between camels, a reservoir species, and humans has been reported but human-to-human transmission in healthcare settings accounts for the majority of cases (4). Clinical presentation commonly includes fever, cough, shortness of breath, myalgia, and diarrhea, while severe cases can progress to acute kidney injury and/or acute respiratory distress syndrome (4, 5). Since 2012, there have been 2,618 confirmed cases of MERS reported in 27 countries, with the majority of these occurring in Saudi Arabia (6). The infection has caused 945 known deaths and it has a case-fatality rate of 36% (6).

Despite the high mortality rate associated with MERS-CoV, no approved therapeutic or prophylactic vaccine currently exists, underscoring a critical unmet medical need. This gap in effective treatment and prevention strategies represents a significant public health concern, particularly in regions where the virus is endemic or poses a future threat (7, 8). While several vaccine candidates are in clinical development, the absence of a licensed product highlights the urgent need for continued innovation in vaccine platforms (9).

DNA-based vaccines have emerged as a promising solution due to their favorable safety profile, ability to elicit both antigen-specific T and B cell responses, thermostability across a wide temperature range, and suitability for rapid, large-scale production (10–13). Unlike viral vector-based platforms, DNA vaccines do not induce anti-vector immunity, thereby allowing for repeated administrations (14, 15).

A growing body of clinical evidence underscores the potential effectiveness and safety of DNA medicines. This approach has demonstrated cellular and humoral immune responses across a range of infectious diseases in both preclinical (16–18) and clinical studies (10, 19–21), in addition to a well-tolerated safety profile. One DNA-based SARS-CoV-2 vaccine (ZyCoV-D) was approved for emergency use in India (22, 23). More relevantly, preclinical (24, 25) and Phase 1 clinical trials (12, 26) have shown that INO-4700, a synthetic DNA-based vaccine composed of a plasmid encoding the full length MERS-CoV spike (S) glycoprotein used in the current study, has a favorable safety and tolerability profile and is immunogenic. In a MERS-CoV challenge using non-human primate models, INO-4700-immunized rhesus macaques exhibited reduced clinical symptoms compared to controls (25).

The primary objectives of this current study were to evaluate the tolerability, safety and immunogenicity of INO-4700 in a demographically relevant population of healthy adult volunteers.

## Materials and methods

### Study design and population

This clinical study was a Phase 2a, randomized, blinded, placebo-controlled, multi-center trial to evaluate INO-4700 administered intradermally (ID) followed by electroporation (EP) to healthy adult volunteers (MERS-201, NCT04588428). The study was conducted at six clinical sites in the Middle East and North Africa (two each in Lebanon, Jordan, and Kenya) selected due to the presence of dromedary camels, the primary reservoir for MERS-CoV, in these regions. The study was performed in accordance with the principles of the Declaration of Helsinki, Good Clinical Practice (GCP), and applicable regulatory requirements. The study was approved by the Lebanon Ministry of Public Health, the Jordan Food and Drug Administration and the Kenya Health Authorities (Pharmacy and Poisons Board (PPB)) as well as all sites’ Ethics Committees. All participants provided written informed consent.

Participants were required to meet the following eligibility criteria: healthy adults ranging in age from 18 to 50; any acceptable chronic medical condition had to be stably managed; able and willing to comply with study procedures; negative serology for Hepatitis B surface antigen (HBsAg), Hepatitis C antibody and Human Immunodeficiency Virus (HIV) antibody; and have no clinically significant electrocardiogram (ECG) findings at screening. Participants had to meet one of the following criteria with respect to reproductive capacity: post-menopausal woman, surgically sterile or have a partner who was sterile, or use of contraception with failure rate of <1% per year.

Key exclusion criteria included the following: pregnancy, breastfeeding or intention to become pregnant or father children; previous recipient of investigational MERS vaccine or any other vaccine (e.g., COVID-19) within 30 days preceding Week 0 or during the restricted timeframe; participating in a study with an investigational product within 30 days preceding Week 0; history of respiratory disease (asthma, chronic obstructive pulmonary disease (COPD), etc.); prior exposure to MERS-CoV or camels; current or anticipated concomitant immunosuppressive therapies prior to dosing; fewer than two acceptable locations for delivery of drug and EP; active drug, alcohol, or substance abuse; involuntary incarceration. Systemic corticosteroids had to be discontinued within three months of first dose of study vaccine.

Participant medical history included all active conditions, and any other past conditions, as well as surgical procedures, which were considered to be clinically significant by the investigator and/or occurred within the 12 weeks prior to screening. Demographic and baseline characteristic data were descriptively summarized. Concomitant medications were recorded.

### Treatment

**Figure 1:**
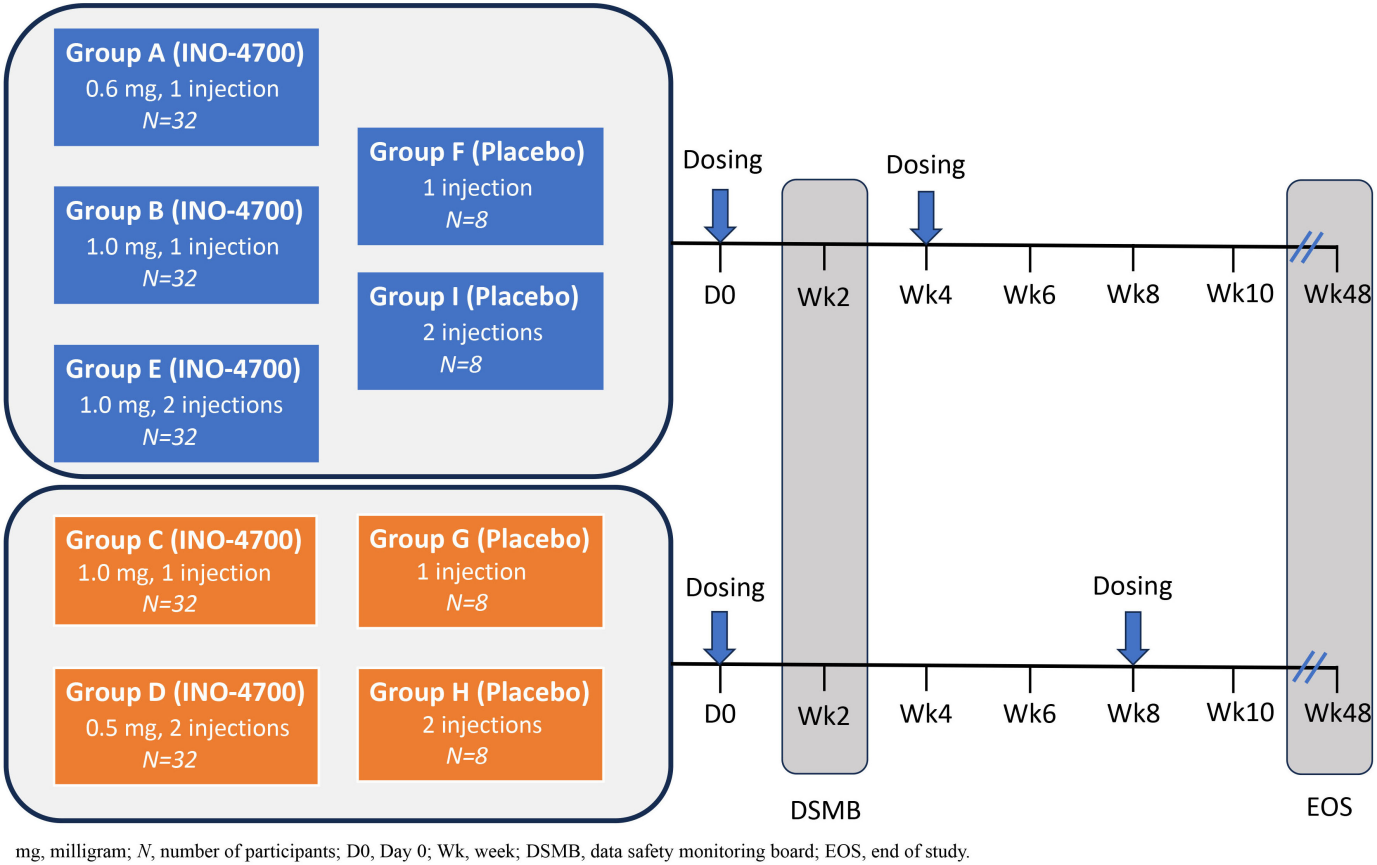
MERS-201 study design. Five dose levels and regimens were evaluated across nine groups. Study Groups A, B, C, D, E received INO-4700 and enrolled 32 participants per group. Study Groups F, G, H, I received placebo (SSC buffer) and enrolled 8 participants per group. INO-4700 or placebo was administered intradermally (ID) into the deltoid area of the upper arms and was followed by electroporation (EP). Study Groups A, B, E, F, and I were dosed at Week 0 and Week 4. Study Groups C, D, G, H were dosed at Week 0 and Week 8. For Groups D, E, H, I, receiving two doses, INO-4700 or placebo were administered in the deltoid of different arms at each dosing visit. The nine study groups were: Group A: INO-4700 1 × 0.6 mg at Week 0 and Week 4. Group B: INO-4700 1 × 1.0 mg at Week 0 and Week 4. Group C: INO-4700 1 × 1.0 mg at Week 0 and Week 8. Group D: INO-4700 2 × 0.5 mg at Week 0 and Week 8. Group E: INO-4700 2 × 1.0 mg at Week 0 and Week 4. Group F: Placebo 1 × 0 mg at Week 0 and Week 4. Group G: Placebo 1 × 0 mg at Week 0 and Week 8. Group H: Placebo 2 × 0 mg at Week 0 and Week 8. Group I: Placebo 2 × 0 mg at Week 0 and Week.

One hundred and ninety-two participants were randomized to receive the intervention (i.e., INO-4700) or placebo via intradermal injection followed by EP across nine study groups (**Table 1**, **Figure 1**). There were 5 intervention groups (Group A through E) and 4 placebo groups (Group F through I). INO-4700 was administered in three-dose levels (0.6 mg, 1 mg, and 2 mg) as one or two ID injections to achieve the total dose level during each of two dosing days. Specifically, INO-4700 groups received 0.6 mg as a single injection, 1 mg (as a single injection or as two 0.5 mg injections in different limbs) and 2 mg (as two 1 mg injections in different limbs) at Week 0 and at either Week 4 or Week 8. Injections were to be administered ID in the deltoid muscles or lateral quadriceps, with the deltoid being the preferred location. Groups receiving two concurrent injections were required to receive each injection in different limbs (different deltoids or lateral quadriceps). Participants were followed up between June 2021 and January 2023.

**Table 1:** Participant disposition in MERS-201 study.

Variable	Intervention groups (INO-4700)					Placebo groups				All groups combined (*N* = 192)
	Group A (*N* = 32)	Group B (*N* = 33)	Group C (*N* = 32)	Group D^a^ (*N* = 32)	Group E^a^ (*N* = 31)	Group F (*N* = 8)	Group G (*N* = 8)	Group H^a^ (*N* = 8)	Group I^a^ (*N* = 8)	
	1 × 0.6 mg at Wks 0, 4	1 × 1.0 mg at Wks 0, 4	1 × 1.0 mg at Wks 0, 8	2 × 0.5 mg at Wks 0, 8	2 × 1.0 mg at Wks 0, 4	1 × 0 mg at Wks 0, 4	1 × 0 mg at Wks 0, 8	2 × 0 mg at Wks 0, 8	2 × 0 mg at Wks 0, 4	
Study treatment, *n* (%)										
Completed	32 (100.0)	32 (97.0)	32 (100.0)	32 (100.0)	31 (100.0)	8 (100.0)	8 (100.0)	8 (100.0)	8 (100.0)	191 (99.5)
Discontinued	0	1 (3.0)	0	0	0	0	0	0	0	1 (0.5)
Study status, *n* (%)										
Completed	32 (100.0)	33 (100.0)	29 (90.6)	31 (96.9)	31 (100.0)	7 (87.5)	8 (100.0)	8 (100.0)	8 (100.0)	187 (97.4)
Discontinued	0	0	3 (9.4)	1 (3.1)	0	1 (12.5)	0	0	0	5 (2.6)
Primary reason for discontinuation, *n* (%)										
Withdrawal by Participant	0	0	1 (3.1)	0	0	1 (12.5)	0	0	0	2 (1.0)
Protocol Deviation	0	0	2 (6.3)	0	0	0	0	0	0	2 (1.0)
Other	0	0	0	1 (3.1)	0	0	0	0	0	1 (0.5)

*N/n*, number of participants; mg, milligram; Wks, weeks.

INO-4700 or placebo was administered intradermally (ID) into the deltoid area of the upper arms and was followed by electroporation (EP).

a. For Groups D, E, H, and I receiving two doses per visit, each dose of INO-4700 or placebo was administered in the deltoid of different arms.

Placebo groups consisted of saline-sodium citrate solution (SSC) in one or two ID injections as a two-dosing-days regimen at Week 0 and at either Week 4 or Week 8. Each ID injection of INO-4700 or placebo had a volume of ~0.1 mL and was followed by EP.

INO-4700 consists of plasmid pGX9101 incorporating a synthetic, optimized microconsensus sequence of the MERS-CoV full length post-fusion spike glycoprotein including multiple conserved epitopes to account for the high mutability of the receptor-binding domain (RBD) and to elicit broad cross-reactive immune responses, as previously described (7, 12). INO-4700 was formulated at a concentration of 10 mg/mL in a saline sodium citrate (SSC) buffer, whereas placebo consisted of SSC buffer only. Both were produced according to current Good Manufacturing Practices and administered ID, followed by EP using Inovio’s proprietary CELLECTRA^®^ 2000 device, which delivers four controlled, brief electrical pulses of 0.2 amps for 52 milliseconds of duration each to enhance local cellular uptake of DNA plasmids by increasing cell membrane permeability (10, 20, 27, 28).

### Objectives and endpoints

The first primary objective of this study was to evaluate the tolerability and safety of INO-4700 in healthy adult volunteers, which is further described below. The second primary objective was to evaluate the cellular and humoral immune responses to INO-4700 based on different doses and dosing schedules. The primary immunogenicity endpoints included overall immune response, as evaluated by MERS-CoV antigen-specific antibodies and antigen-specific cytokine-producing T cell responses. Exploratory objectives/endpoints included assessment of SARS-CoV-2 cross-reactivity and potential impact on INO-4700 seroconversion, by evaluating participant sera for SARS-CoV-2 spike and nucleocapsid protein (NP)-binding IgG.

#### Safety evaluations

The primary safety endpoints of the study included: the incidence of adverse events (AEs), the frequency and severity of injection-site reactions, and the incidence of AEs of special interest (AESIs). The full safety analyses included the assessment of treatment-emergent adverse events (TEAEs), their toxicity grading scale (Grade 1 through Grade 4), in accordance with the Toxicity Grading Scale for Healthy Adult and Adolescent Volunteers Enrolled in Preventive Vaccine Clinical Trials guidelines for industry (29), seriousness (serious adverse events [SAEs]), their relationship to treatment (treatment-related TEAEs [TR-TEAEs]) and AEs of special interest (AESIs).

AESIs relevant to development of a MERS-CoV vaccine that were monitored for during the study period included, but were not limited to, COVID-19 infection, thrombocytopenia, pneumonitis, and acute respiratory distress syndrome (ARDS). Participants were followed through Week 48.

#### Immunological assessments

Whole blood and serum samples were obtained at Screening, Week 0, Week 6 and Week 10 to assess immune responses. Humoral and cellular immune responses to INO-4700 were measured by assays for MERS-CoV antigen-specific binding antibodies, MERS-CoV neutralizing antibodies, and antigen-specific cytokine-producing T-cells. Additionally, assays for binding antibodies to SARS-CoV-2 were used to assess their potential impact on INO-4700 seroconversion rates, as the study was conducted during the SARS-CoV-2 pandemic.

Humoral and cellular immune responses were analyzed without exclusion (as per the Protocol) or with exclusion of SARS-CoV-2 convalescent vaccinated participants as determined by SARS-CoV-2 spike protein or SARS-CoV-2 NP IgG seropositivity.

##### Antigen-specific IgG binding antibody assays

Binding IgG antibodies specific to the RBD region of the MERS-CoV spike protein were measured in study serum samples using a Meso Scale Discovery electrochemiluminescent (MSD ECL) assay developed at Inovio Pharmaceuticals. Standard MULTI-ARRAY MSD plates (Meso Scale Discovery, Rockville, MD) were coated overnight with 50 µL of recombinant MERS-CoV spike RBD protein (ACROBiosystems, Beijing, China) at 4 µg/mL. Plates were washed three times in wash buffer consisting of 1x PBS with 0.05% Tween-20 (Sigma-Aldrich, Burlington, MA) prior to each of the following steps: plates were blocked with 300 uL of Candor BSA Block (Boca Scientific, Dedham, MA) for 1–3 hours; 30 µL per well of study serum samples were diluted 1/100 in Blocker Casein in PBS (ThermoFischer Scientific, Waltham, MA), added to the plates then incubated on a plate shaker at 600 RPM for one hour; 30 µL per well of 0.25x SULFO-TAG anti-human IgG antibody (Meso Scale Discovery) was added and incubated for one hour on a plate shaker at 600 RPM; 100 µL of 1x MSD Read Buffer T was added per well followed by signal acquisition using an MSD sector S 6000 microplate imager (Meso Scale Discovery). The concentration of anti-MERS-CoV spike RBD antibody in study samples was determined by interpolation from a 7-point standard curve of serially diluted recombinant human Anti-MERS-CoV S antibody MCA1 (Creative Biolabs, Shirley, NY) using dilutions ranging from 1/10,000 to 1/36,450,000. The standard curve antibody was calibrated to the 1st World Health Organization (WHO) International Standard for Anti-MERS-CoV Immunoglobulin G (30), and antibody concentrations were expressed in International Units per mL (IU/mL).

##### Assessment of antibodies to MERS-CoV full-length spike protein and SARS-CoV-2 spike and nucleocapsid proteins

A multiplexed assay kit, V-PLEX COVID-19 Coronavirus Panel 3 (IgG) Kit (Mesoscale Discovery), was adapted for the quantitative measurement of antibodies to MERS-CoV full length spike (S) protein. The assay was performed as instructed by the manufacturer except for the inclusion of an additional seven-point standard curve of serially diluted recombinant human anti-MERS-CoV S antibody MCA1 (Creative Biolabs), calibrated to the 1st WHO International Standard for Anti-MERS-CoV Immunoglobulin G (30), to quantify the concentration of MERS-CoV spike antibody in IU/mL. The standard curve included in the kit was used to quantify the concentration of antibodies to SARS-CoV-2 spike and nucleocapsid protein and to determine SARS-CoV-2 seropositivity using manufacturer-suggested seropositivity cutoffs, which were established using receiver operating characteristic (ROC) curves obtained using panels of serum acquired prior to the SARS-CoV-2 pandemic and serum from individuals with PCR-confirmed COVID-19.

##### Antigen-specific cytokine-producing T cell responses

To detect cytokine-secreting T cells in participant peripheral blood mononuclear cells (PBMCs), Interferon-gamma (IFN-γ) Enzyme-Linked ImmunoSpot (ELISpot) assays were performed using the Human Interferon-γ ELISpot Pro kit (Mabtec, Nacka Strand, Sweden) with 15-mer peptides comprising the full sequence of MERS-CoV spike protein with an overlap of nine amino acids (JPT Peptide Technologies, Berlin, Germany). Peptides were split into three separate pools encompassing the N-terminal, central and C-terminal regions of MERS-CoV spike and added to each well to a final concentration of 2 μg/mL per peptide. Three x 10^5^ PBMCs were added per well in triplicate and stimulated with peptides for 18–24 hours prior to colorimetric spot development of assay plates. Spots were enumerated using Immunospot software (Immunospot, Shaker Heights, Cleveland, OH), and data was reported as the mean spot forming units (SFUs) per million PBMCs after subtracting the background response obtained from negative control dimethyl sulfoxide (DMSO)-stimulated wells.

##### MERS spike pseudotyped lentiviral neutralization assay

A MERS spike pseudotyped lentiviral neutralization assay was developed and performed at Guard Rx (Trois-Rivières, QC, Canada). To produce the MERS spike Luc-2 pseudovirus, one mL of polyethylenimine (PEI)/DNA solution was prepared by combining 4 µg pHAGE-CMV-Luc2-IRES-zsGreen (lentiviral transfer vector/reporter gene), 3 µg psPAX2 (packaging plasmid), 3 µg of pCMV-MERS-spike delta14 (MERS-CoV spike glycoprotein with cytoplasmic tail deletion) and 45 µL of PEI. After incubating for 20 minutes at room temperature, 1 mL of the solution was added per 10 cm dish containing 70-80% confluent HEK293T cells and incubated overnight at 37 °C with 5% CO_2_. Pseudovirus-containing supernatants were harvested 48 to 72 hours post-transfection, filtered through a 0.45 µm filter, aliquoted and frozen.

For the pseudoneutralization assay, 3 x 10^4^ HEK293 cells expressing DPP4 in 100 µL per well of complete Dulbecco’s Modified Eagle Medium (DMEM) containing 10% FBS (heat inactivated, qualified, Gibco OneShot FBS, ThermoFischer Scientific) and 1x Pen-Strep (Corning, Corning, NY) were seeded per well of a white 96 well plate (Greiner Bio-One, Kremsmünster, Austria) and incubated overnight at 37 °C with 5% CO_2_. 200 µL of a six-point, two-fold serial dilution series (starting from 1/20) of study sample serum, was mixed with 100 uL of MERS-spike Luc-2 pseudovirus and incubated at room temperature for 30 minutes before adding to HEK293-DPP4 cells. Cells were incubated with pseudovirus for 72 hours at 37 °C with 5% CO_2_ prior to quantitation of luciferase expression, which involved adding 100 µL per well of Bright-Glo (Promega, Madison, WI) and reading luminescence using a BioTek Synergy HT Plate Reader (Agilent, Santa Clara, California). Fifty percent inhibitory dose (ID_50_) values were calculated by fitting data with a three-parameter logistic regression model using GraphPad Prism software (San Diego, California).

### Statistical and data analyses

The participant disposition was summarized for all randomized participants and included the number and percentage randomized, the number and percentage who received each planned dose and the number who completed each part of the trial. Demographics and baseline characteristics were summarized descriptively. Missing data were not imputed or replaced. No formal power analysis was applicable in this study.

The safety analysis population consisted of all participants who received at least one dose of INO-4700 or placebo and were grouped in accordance with the dose of INO-4700 or placebo received. The modified intent-to-treat (mITT) analysis population also consisted of all participants who received at least one dose of INO-4700 or placebo and were analyzed by their original assigned dose of INO-4700 or placebo.

All safety analyses were conducted using the safety analysis population and tabulations were provided by study group.

Immunogenicity analyses, including co-primary and exploratory immunological endpoints, including humoral and cellular immune responses to INO-4700 were conducted on participants in the mITT analysis population. A MERS RBD binding responder or MERS spike binding IgG responder was defined as a participant with a post-treatment concentration that was greater than 4 times the baseline value. A MERS spike ELISpot responder was defined as a participant with a post-treatment level that was greater than baseline plus 2 standard deviations plus the assay limit of quantitation (LOQ).

## Results

### Participant demographics and baseline characteristics

**Figure 2:**
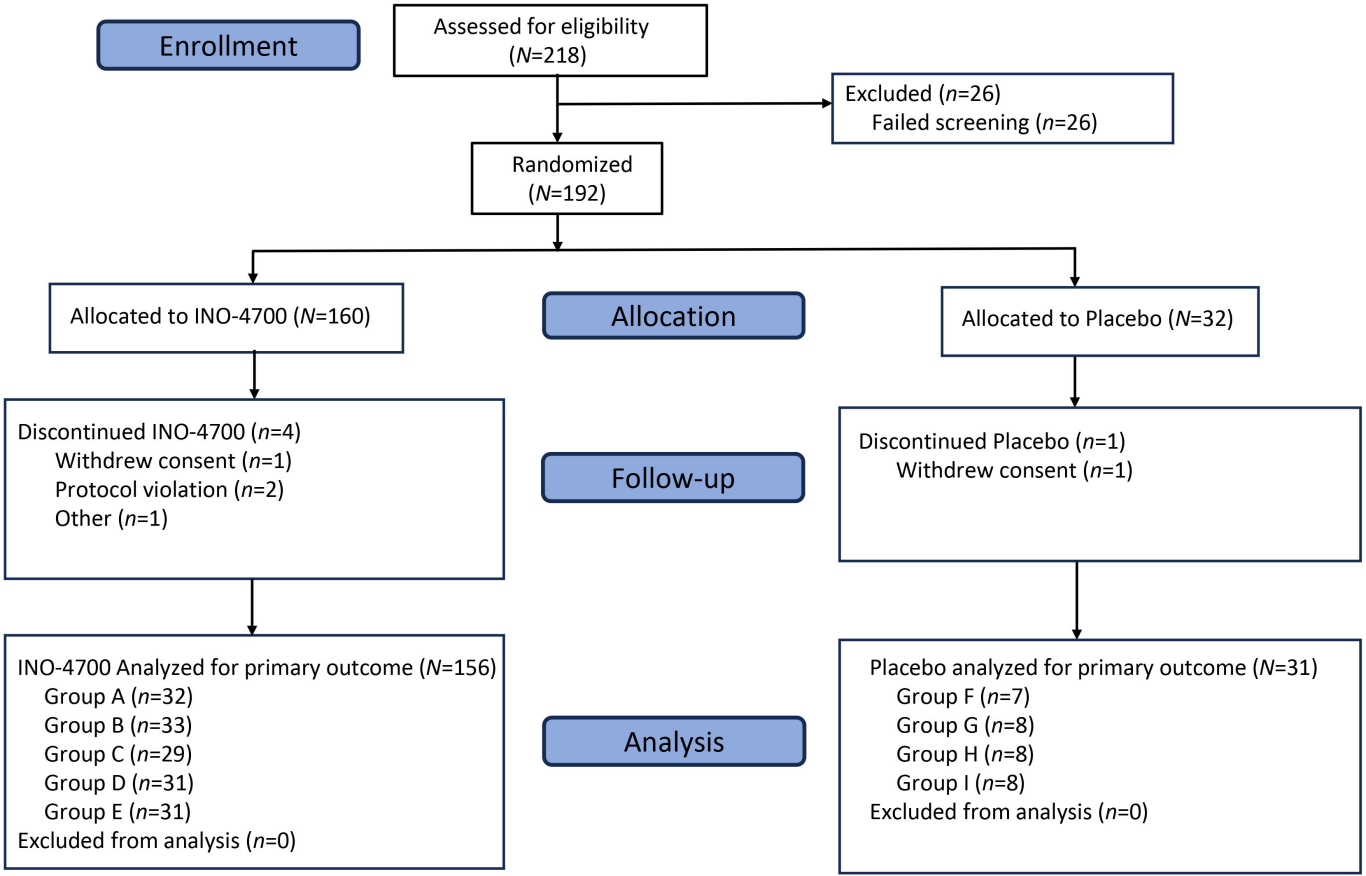
CONSORT diagram. MERS-201 was a Phase 2a, randomized, blinded, placebo-controlled, multi-center trial enrolling participants at a ratio of 4:1 to receive INO-4700 or placebo. Eligibility was assessed for 218 subjects and 192 participants were enrolled across nine groups (Groups A–I). Of the 192 enrolled, 187 participants completed the trial. *N/n*, number of participants.

A total of 218 participants were screened, with 192 subsequently enrolled and randomized to either an INO-4700 (*n* = 160) or placebo group (*n* = 32) (**Figure 2**). All participants received the ID injections in the deltoid area of the upper arms followed by EP. Participants assigned to INO-4700 groups received at least one dose of the investigational DNA vaccine. All but one participant (*n* = 191, 99.5%) completed treatment. Among the participants enrolled, 187 (97.4%) completed all study visits (**Table 1**). Overall, the average age of the participants was 33.8 years. Most were male (64.6%) and white (89.6%) (**Table 2**).

**Table 2:** Demographics and other baseline characteristics in MERS-201 study.

Variable	Intervention groups (INO-4700)						Placebo groups					All groups combined (*N* = 192)
	Group A (*N* = 32)	Group B (*N* = 33)	Group C (*N* = 32)	Group D^a^ (*N* = 32)	Group E^a^ (*N* = 31)	Total Intervention (*N* = 160)	Group F (*N* = 8)	Group G (*N* = 8)	Group H^a^ (*N* = 8)	Group I^a^ (*N* = 8)	Total placebo (*N* = 32)	
	1 × 0.6 mg at Wks 0, 4	1 × 1.0 mg at Wks 0, 4	1 × 1.0 mg at Wks 0, 8	2 × 0.5 mg at Wks 0, 8	2 × 1.0 mg at Wks 0, 4		1 × 0 mg at Wks 0, 4	1 × 0 mg at Wks 0, 8	2 × 0 mg at Wks 0, 8	2 × 0 mg at Wks 0, 4		
Age (years)												
Mean (SD)	34.4 (7.66)	34.2 (9.20)	34.8 (7.69)	31.5 (8.63)	35.0 (7.77)	34.0 (8.22)	28.4 (8.40)	34.0 (10.62)	30.3 (7.52)	39.8 (6.96)	33.1 (9.20)	33.8 (8.37)
Median	34.5	35	35.5	34.5	37	35	24.5	30.5	28	41	33	35
Min, Max	20, 47	18, 48	20, 48	19, 49	19, 47	18, 49	20, 42	23, 49	23, 44	24, 47	20, 49	18, 49
Sex, *n* (%)												
Female	10 (31.3)	12 (36.4)	11 (34.4)	10 (31.3)	16 (51.6)	59 (36.9)	0	4 (50.0)	3 (37.5)	2 (25.0)	9 (28.1)	68 (35.4)
Male	22 (68.8)	21 (63.6)	21 (65.6)	22 (68.8)	15 (48.4)	101 (63.1)	8 (100.0)	4 (50.0)	5 (62.5)	6 (75.0)	23 (71.9)	124 (64.6)
Race, *n* (%)												
White	30 (93.8)	28 (84.8)	26 (81.3)	30 (93.8)	29 (93.5)	143 (89.4)	7 (87.5)	7 (87.5)	7 (87.5)	8 (100.0)	29 (90.6)	172 (89.6)
Black or African American	2 (6.3)	5 (15.2)	6 (18.8)	2 (6.3)	2 (6.5)	17 (10.6)	1 (12.5)	1 (12.5)	1 (12.5)	0 (0.0)	3 (9.4)	20 (10.4)
BMI (kg/m^2^)												
Mean (SD)	28.04 (4.75)	25.74 (4.18)	26.51 (4.76)	26.52 (4.49)	29.28 (5.94)	27.20 (4.95)	26.08 (3.36)	29.27 (5.91)	29.03 (5.47)	26.36 (4.11)	27.68 (4.82)	27.28 (4.93)
Median	27.339	25.654	26.33	26.803	28.387	26.574	25.71	30.566	27.977	27.055	27.657	26.842
Min, Max	18.49, 39.55	16.66, 34.78	18.72, 39.26	18.59, 36.50	19.59, 39.96	16.66, 39.96	19.94, 31.12	17.45, 36.7	21.97, 38.71	20.32, 32.32	17.45, 38.71	16.66, 39.96

*N/n*, number of participants; mg, milligram; Wks, weeks; SD, standard deviation; max, maximum; min, minimum; BMI, body mass index; kg, kilograms; m, meters.

INO-4700 or placebo was administered intradermally (ID) into the deltoid area of the upper arms and was followed by electroporation (EP).

a. For Groups D, E, H, and I receiving two doses per visit, each dose of INO-4700 or placebo was administered in the deltoid of different arms.

### Safety assessments

Treatment with INO-4700 administered ID followed by EP was well-tolerated and had a favorable safety profile with low incidence of TEAEs, with most of them assessed as Grade 1 or Grade 2, non-serious, and unrelated to treatment.

Specifically, in the Safety Population, 72 (37.5%) participants experienced a total of 130 TEAEs (**Table 3**), (36.3% vs. 43.8% in INO-4700 and placebo participants, respectively). 50 (26%), 31 (16%) and 4 (2.1%) of all participants experienced a total of 79 Grade 1, 43 Grade 2, and 6 Grade 3 TEAEs. Two participants (1.0%) experienced a single Grade 4 TEAE. All Grade 3 and Grade 4 TEAEs were assessed as not related to treatment. The most frequent TEAEs reported were hyperglycemia (6.3% vs. 6.3%), increased blood glucose (5.0% vs. 6.3%) and headache (5.0% vs. 6.3%) in the INO-4700 and placebo groups respectively (**Table 4**). Of the 130 TEAEs, 17 events were assessed as treatment-related TEAEs (TR-TEAEs) in only 10 (5.2%) participants. Among them, 9 (5.6%) participants received INO-4700 and 1 (3.1%) participant received placebo (**Table 4**). All TR-TEAEs were Grade 1 or 2 in severity. All injection site reactions were assessed as Grade 1 in severity. The most commonly reported TR-TEAEs included headache (6 participants, 3.1%), fatigue (3 participants, 1.6%) and injection site pruritus (3 participants, 1.6%). All other TR-TEAEs were reported in one participant each: injection site erythema, dizziness, and muscle spasms.

**Table 3:** Summary of adverse events in MERS-201 study safety population.

Variable	Intervention groups (INO-4700)												Placebo groups										All groups combined (*N* = 192)	
	Group A (*N* = 32)		Group B (*N* = 33)		Group C (*N* = 32)		Group D^a^ (*N* = 32)		Group E^a^ (*N* = 31)		Total intervention (*N* = 160)		Group F (*N* = 8)		Group G (*N* = 8)		Group H^a^ (*N* = 8)		Group I^a^ (*N* = 8)		Total placebo (*N* = 32)			
	1 × 0.6 mg at Wks 0, 4		1 × 1.0 mg at Wks 0, 4		1 × 1.0 mg at Wks 0, 8		2 × 0.5 mg at Wks 0, 8		2 × 1.0 mg at Wks 0, 4				1 × 0 mg at Wks 0, 4		1 × 0 mg at Wks 0, 8		2 × 0 mg at Wks 0, 8		2 × 0 mg at Wks 0, 4					
	*n* (%)	Events	*n* (%)	Events	*n* (%)	Events	*n* (%)	Events	*n* (%)	Events	*n* (%)	Events	*n* (%)	Events	*n* (%)	Events	*n* (%)	Events	*n* (%)	Events	*n* (%)	Events	*n* (%)	Events
Subjects with ≥1 Adverse Events (AE)	11 (34.4)	16	12 (36.4)	25	14 (43.8)	40	11 (34.4)	16	13 (41.9)	20	61 (38.1)	117	2 (25.0)	2	6 (75.0)	9	2 (25.0)	5	4 (50.0)	4	14 (43.8)	20	75 (39.1)	137
Subjects with at Least One Pre-treatment AE	0 (0.0)	0	3 (9.1)	3	0 (0.0)	0	1 (3.1)	1	2 (6.5)	3	6 (3.8)	7	0 (0.0)	0	0 (0.0)	0	0 (0.0)	0	0 (0.0)	0	0 (0.0)	0	6 (3.1)	7
Subjects with ≥1 Treatment-emergent AE (TEAE)	11 (34.4)	16	10 (30.3)	22	14 (43.8)	40	10 (31.3)	15	13 (41.9)	17	58 (36.3)	110	2 (25.0)	2	6 (75.0)	9	2 (25.0)	5	4 (50.0)	4	14 (43.8)	20	72 (37.5)	130
Subjects with ≥1 Serious AE (SAE)	0 (0.0)	0	1 (3.0)	1	0 (0.0)	0	1 (3.1)	1	0 (0.0)	0	2 (1.3)	2	0 (0.0)	0	0 (0.0)	0	0 (0.0)	0	0 (0.0)	0	0 (0.0)	0	2 (1.0)	2
Subjects with ≥1 Grade 1 TEAE	7 (21.9)	11	8 (24.2)	13	9 (28.1)	21	9 (28.1)	13	10 (32.3)	11	43 (26.9)	69	0 (0.0)	0	4 (50.0)	5	1 (12.5)	3	2 (25.0)	2	7 (21.9)	10	50 (26.0)	79
Subjects with ≥1 Grade 2 TEAE	4 (12.5)	4	4 (12.1)	8	9 (28.1)	14	1 (3.1)	2	4 (12.9)	5	22 (13.8)	33	2 (25.0)	2	4 (50.0)	4	1 (12.5)	2	2 (25.0)	2	9 (28.1)	10	31 (16.1)	43
Subjects with ≥1 Grade 3 TEAE	1 (3.1)	1	0 (0.0)	0	2 (6.3)	4	0 (0.0)	0	1 (3.2)	1	4 (2.5)	6	0 (0.0)	0	0 (0.0)	0	0 (0.0)	0	0 (0.0)	0	0 (0.0)	0	4 (2.1)	6
Subjects with ≥1 Grade 4 TEAE	0 (0.0)	0	1 (3.0)	1	1 (3.1)	1	0 (0.0)	0	0 (0.0)	0	2 (1.3)	2	0 (0.0)	0	0 (0.0)	0	0 (0.0)	0	0 (0.0)	0	0 (0.0)	0	2 (1.0)	2
Subjects with ≥1 TEAE leading to Death	0 (0.0)	0	0 (0.0)	0	0 (0.0)	0	0 (0.0)	0	0 (0.0)	0	0 (0.0)	0	0 (0.0)	0	0 (0.0)	0	0 (0.0)	0	0 (0.0)	0	0 (0.0)	0	0 (0.0)	0
Subjects with ≥1 TEAE leading to Treatment Discontinuation	0 (0.0)	0	0 (0.0)	0	0 (0.0)	0	0 (0.0)	0	0 (0.0)	0	0 (0.0)	0	0 (0.0)	0	0 (0.0)	0	0 (0.0)	0	0 (0.0)	0	0 (0.0)	0	0 (0.0)	0
Subjects with ≥1 AE of Special Interest (AESI)	0 (0.0)	0	2 (6.1)	2	1 (3.1)	1	0 (0.0)	0	2 (6.5)	2	5 (3.1)	5	0 (0.0)	0	0 (0.0)	0	1 (12.5)	1	0 (0.0)	0	1 (3.1)	1	6 (3.1)	6
Subjects with ≥1 Treatment-related TEAE (TR-TEAE)	1 (3.1)	1	3 (9.1)	6	1 (3.1)	1	3 (9.4)	7	1 (3.2)	1	9 (5.6)	16	0 (0.0)	0	0 (0.0)	0	0 (0.0)	0	1 (12.5)	1	1 (3.1)	1	10 (5.2)	17
Subjects with ≥1 Serious TR-TEAE	0 (0.0)	0	0 (0.0)	0	0 (0.0)	0	0 (0.0)	0	0 (0.0)	0	0 (0.0)	0	0 (0.0)	0	0 (0.0)	0	0 (0.0)	0	0 (0.0)	0	0 (0.0)	0	0 (0.0)	0

*N/n*, number of participants; mg, milligram; Wks, weeks; AE, adverse event; TEAE, treatment-emergent adverse event; SAE, serious adverse event; TESAE, treatment-emergent serious adverse event; AESI, adverse event of special interest; TR-TEAE, treatment-related treatment-emergent adverse event.

A TEAE was defined as any AE that occurs within 30 days of the last treatment.

INO-4700 or placebo was administered intradermally (ID) into the deltoid area of the upper arms and was followed by electroporation (EP).

a. For Groups D, E, H, and I receiving two doses per visit, each dose of INO-4700 or placebo was administered in the deltoid of different arms.

**Table 4:** Treatment-emergent adverse events occurring in ≥ 2% of participants by preferred term and relation to treatment in the MERS-201 study safety population.

Type of event/preferred term	Intervention groups (INO-4700)						Placebo groups					All groups combined (*N* = 192)
	Group A (*N* = 32)	Group B (*N* = 33)	Group C (*N* = 32)	Group D^a^ (*N* = 32)	Group E^a^ (*N* = 31)	Total intervention (*N* = 160)	Group F (*N* = 8)	Group G (*N* = 8)	Group H^a^ (*N* = 8)	Group I^a^ (*N* = 8)	Total placebo (*N* = 32)	
	1 × 0.6 mg at Wks 0, 4	1 × 1.0 mg at Wks 0, 4	1 × 1.0 mg at Wks 0, 8	2 × 0.5 mg at Wks 0, 8	2 × 1.0 mg at Wks 0, 4		1 × 0 mg at Wks 0, 4	1 × 0 mg at Wks 0, 8	2 × 0 mg at Wks 0, 8	2 × 0 mg at Wks 0, 4		
	*n* (%)	*n* (%)	*n* (%)	*n* (%)	*n* (%)	*n* (%)	*n* (%)	*n* (%)	*n* (%)	*n* (%)	*n* (%)	*n* (%)
Total number of treatment-emergent AE (TEAE)	16	22	40	15	17	110	2	9	5	4	20	130
Subjects witd ≥1 TEAE	11 (34.4)	10 (30.3)	14 (43.8)	10 (31.3)	13 (41.9)	58 (36.3)	2 (25.0)	6 (75.0)	2 (25.0)	4 (50.0)	14 (43.8)	72 (37.5)
Headache	0 (0.0)	4 (12.1)	1 (3.1)	1 (3.1)	2 (6.5)	8 (5.0)	0 (0.0)	1 (12.5)	0 (0.0)	1 (12.5)	2 (6.3)	10 (5.2)
COVID-19	0 (0.0)	2 (6.1)	2 (6.3)	0 (0.0)	1 (3.2)	5 (3.1)	0 (0.0)	0 (0.0)	1 (12.5)	0 (0.0)	1 (3.1)	6 (3.1)
Urinary tract infection	0 (0.0)	0 (0.0)	2 (6.3)	0 (0.0)	3 (9.7)	5 (3.1)	0 (0.0)	1 (12.5)	0 (0.0)	0 (0.0)	1 (3.1)	6 (3.1)
Pharyngitis	0 (0.0)	2 (6.1)	3 (9.4)	0 (0.0)	0 (0.0)	5 (3.1)	0 (0.0)	0 (0.0)	0 (0.0)	0 (0.0)	0 (0.0)	5 (2.6)
Fatigue	2 (6.3)	1 (3.0)	0 (0.0)	1 (3.1)	0 (0.0)	4 (2.5)	0 (0.0)	0 (0.0)	0 (0.0)	0 (0.0)	0 (0.0)	4 (2.1)
Pyrexia	1 (3.1)	1 (3.0)	2 (6.3)	0 (0.0)	0 (0.0)	4 (2.5)	0 (0.0)	0 (0.0)	0 (0.0)	0 (0.0)	0 (0.0)	4 (2.1)
Upper respiratory tract infection	0 (0.0)	2 (6.1)	2 (6.3)	0 (0.0)	0 (0.0)	4 (2.5)	0 (0.0)	0 (0.0)	0 (0.0)	0 (0.0)	0 (0.0)	4 (2.1)
Laboratory:												
Hyperglycemia	2 (6.3)	2 (6.1)	3 (9.4)	1 (3.1)	2 (6.5)	10 (6.3)	0 (0.0)	1 (12.5)	0 (0.0)	1 (12.5)	2 (6.3)	12 (6.3)
Blood glucose increased	0 (0.0)	1 (3.0)	0 (0.0)	4 (12.5)	3 (9.7)	8 (5.0)	0 (0.0)	1 (12.5)	0 (0.0)	1 (12.5)	2 (6.3)	10 (5.2)
Alanine aminotransferase increased	1 (3.1)	0 (0.0)	2 (6.3)	0 (0.0)	0 (0.0)	3 (1.9)	0 (0.0)	0 (0.0)	1 (12.5)	0 (0.0)	1 (3.1)	4 (2.1)
Aspartate aminotransferase increased	1 (3.1)	0 (0.0)	2 (6.3)	0 (0.0)	0 (0.0)	3 (1.9)	0 (0.0)	0 (0.0)	1 (12.5)	0 (0.0)	1 (3.1)	4 (2.1)
Blood bicarbonate decreased	0 (0.0)	0 (0.0)	2 (6.3)	0 (0.0)	1 (3.2)	3 (1.9)	1 (12.5)	0 (0.0)	0 (0.0)	0 (0.0)	1 (3.1)	4 (2.1)
Total Number of All treatment-related TEAE (TR-TEAE)	1	6	1	7	1	16	0	0	0	1	1	17
Subjects with ≥1 TR-TEAE	1 (3.1)	3 (9.1)	1 (3.1)	3 (9.4)	1 (3.2)	9 (5.6)	0 (0.0)	0 (0.0)	0 (0.0)	1 (12.5)	1 (3.1)	10 (5.2)
Headache	0 (0.0)	3 (9.1)	0 (0.0)	1 (3.1)	1 (3.2)	5 (3.1)	0 (0.0)	0 (0.0)	0 (0.0)	1 (12.5)	1 (3.1)	6 (3.1)
Fatigue	1 (3.1)	1 (3.0)	0 (0.0)	1 (3.1)	0 (0.0)	3 (1.9)	0 (0.0)	0 (0.0)	0 (0.0)	0 (0.0)	0 (0.0)	3 (1.6)
Injection site pruritus	0 (0.0)	1 (3.0)	0 (0.0)	2 (6.3)	0 (0.0)	3 (1.9)	0 (0.0)	0 (0.0)	0 (0.0)	0 (0.0)	0 (0.0)	3 (1.6)
Injection site erythema	0 (0.0)	0 (0.0)	0 (0.0)	1 (3.1)	0 (0.0)	1 (0.6)	0 (0.0)	0 (0.0)	0 (0.0)	0 (0.0)	0 (0.0)	1 (0.5)
Dizziness	0 (0.0)	1 (3.0)	0 (0.0)	0 (0.0)	0 (0.0)	1 (0.6)	0 (0.0)	0 (0.0)	0 (0.0)	0 (0.0)	0 (0.0)	1 (0.5)
Muscle spasms	0 (0.0)	0 (0.0)	1 (3.1)	0 (0.0)	0 (0.0)	1 (0.6)	0 (0.0)	0 (0.0)	0 (0.0)	0 (0.0)	0 (0.0)	1 (0.5)

*N/n*, number of participants; mg, milligram; Wks, weeks; TEAE, treatment-emergent adverse event; COVID-19, coronavirus disease 2019; TR-TEAE, treatment-related treatment-emergent adverse event.

A TEAE was defined as any AE that occurs within 30 days of the last treatment.

INO-4700 or placebo was administered intradermally (ID) into the deltoid area of the upper arms and was followed by electroporation (EP).

a. For Groups D, E, H and I receiving two doses of INO-4700 per visit, each dose was administered in the deltoid of different arms.

Two participants (1.0%) experienced one SAE each (**Supplementary Table S1**). Six participants (3.1%) experienced AESIs, including 5 subjects in the INO-4700 group (3.1%) and one subject in the placebo group (3.1%). Reported AESIs were COVID-19 (*n* = 4), thrombocytopenia (*n* = 1) and pneumonia (*n* = 1). All SAEs and AESIs were deemed unrelated to study treatment. None of the AEs led to discontinuation or death. The overall safety findings indicated a favorable tolerability profile for INO-4700 (**Table 3**).

### Humoral immune response

INO-4700 administration induced humoral immune responses demonstrated by significant increases above baseline in RBD-specific IgG antibodies across all five groups at Weeks 6 and 10, as assessed by MSD ECL assay (**Supplementary Table S2**). This included two groups (C and D) that had only received a single dose by Week 6. Following the second dose at Week 8, both Groups C and D showed another significant increase in RBD-specific IgG levels above baseline at Week 10 compared to Week 6, whereas Groups A, B, and E, who received their second dose at Week 4, did not show a significant increase from Week 6 to Week 10. The combined placebo group showed no significant increases above baseline in RBD-specific IgG levels between any timepoints (**Supplementary Table S2**).

**Figure 3:**
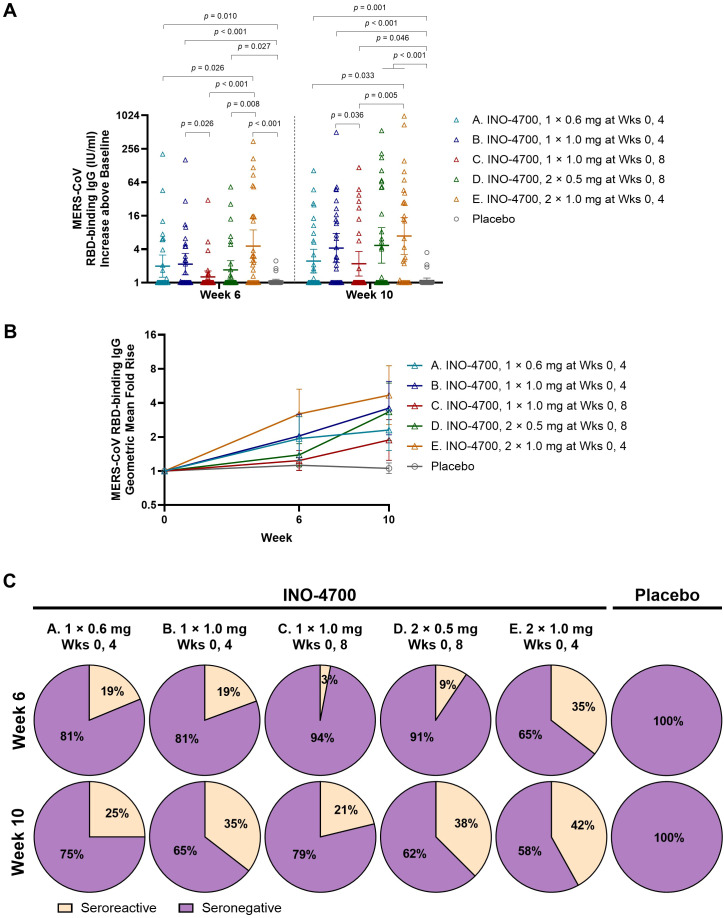
MERS-CoV spike RBD-specific antibody levels as measured by MSD ECL assay. **(A)**. MERS-CoV RBD-binding IgG concentrations (IU/mL) above baseline are shown for each by group. Geometric mean is indicated by a horizontal line with whiskers representing 95% CI. *p*-values were calculated between groups within each timepoint using Wilcoxon rank-sum (Mann-Whitney U) test. Only significant *p*-values are shown. **(B)** Geometric Mean Fold Rise (GMFR) of MERS-CoV spike RBD-specific binding IgG concentrations (IU/mL) from baseline are shown at Weeks 6 and 10. Open symbols represent the GMFR for each study group, whiskers represent the 95% CI. **(C)** Percent seroreactive participants with four-fold or greater increase in MERS-CoV spike RBD-specific binding IgG concentration (IU/mL) from baseline per group. Placebo groups are combined. MERS-CoV, Middle East respiratory syndrome coronavirus; RBD, receptor binding domain; MSD ECL, Meso Scale Discovery electrochemiluminescence; IgG, immunoglobulin G; IU, international units; mL, milliliters; CI, confidence interval; mg, milligram; Wks, weeks.

At Week 10, all INO-4700-treated groups exhibited significantly greater increases in MERS-CoV RBD-specific IgG levels above baseline compared to the combined placebo group (**Figure 3A**). Similar results were observed at Week 6. Differences between INO-4700 regimens were also apparent. At Week 6, Group E, which received the largest cumulative dose of 2.0 mg INO-4700 at both Weeks 0 and 4, had higher antibody levels above baseline relative to all other INO-4700 groups, and significantly higher levels relative to all but Group B. This trend continued into Week 10. At Week 6, Group B, which received two doses of 1 mg INO-4700, had significantly higher antibody levels than Group C, which had received only the first dose of 1.0 mg INO-4700. This significant difference continued through Week 10, despite Group C having received the second dose. Lastly, although Group B received a higher dose of INO-4700 (1.0 mg) than Group A (0.6 mg) injected at a single site on the same dosing schedule, both groups exhibited comparable RBD-specific IgG levels above baseline at all measured timepoints.

INO-4700 immunization led to consistent increases in the geometric mean fold rise (GMFR) of MERS-CoV RBD-specific IgG levels across all groups, from baseline through Week 10 (**Figure 3B**). By Week 10, Group E had the greatest GMFR (4.69), while the GMFR of the placebo group showed little change at 1.06. The rate of seroreactivity to MERS-CoV RBD was also determined (**Figure 3C**). For all INO-4700 groups, the rate of seroreactivity peaked at Week 10 and ranged from 21% for Group C up to 42% for Group E. All participants in the placebo group remained seronegative.

**Figure 4:**
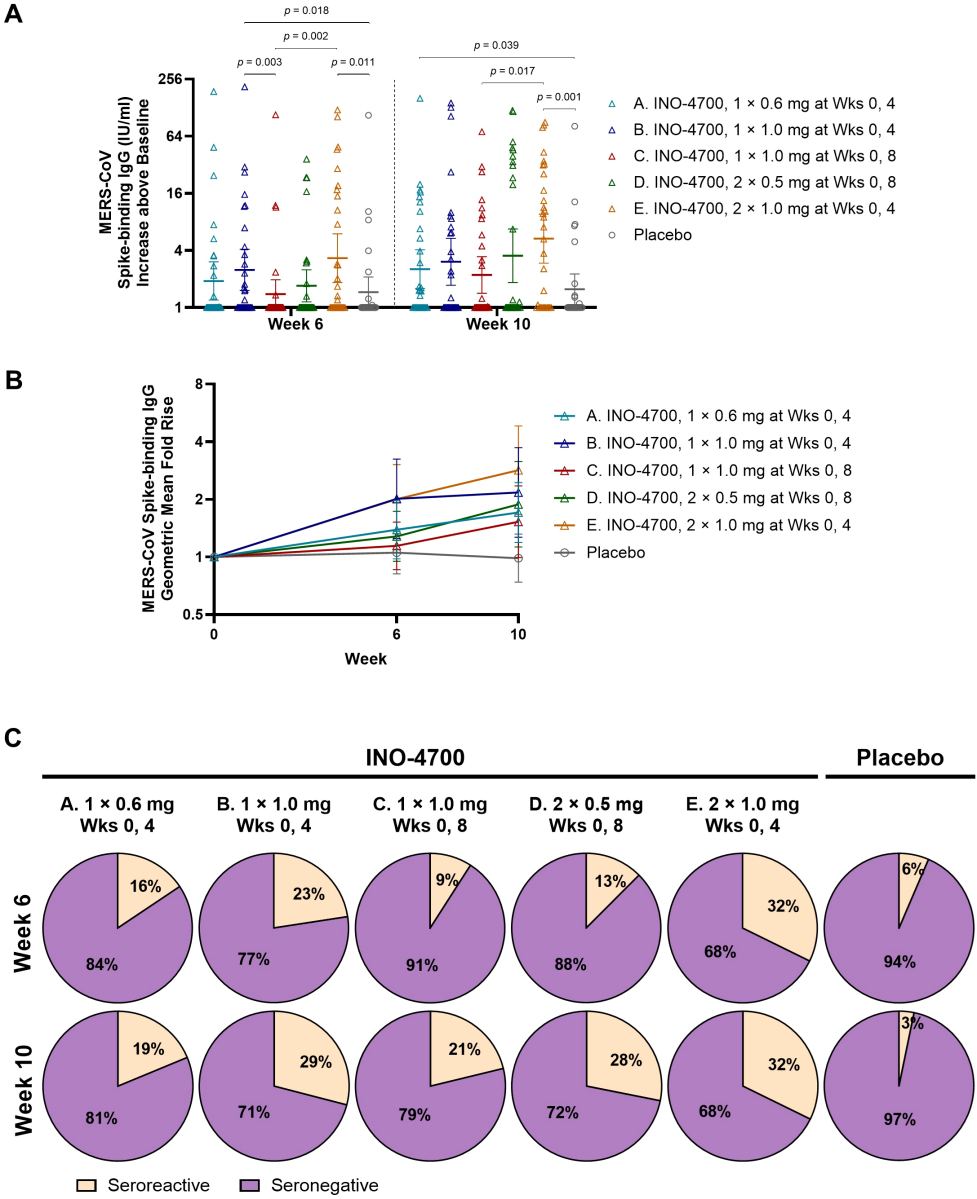
MERS-CoV spike-specific antibody levels as measured by MSD ECL assay. **(A)** MERS-CoV spike-binding IgG concentrations (IU/mL) above baseline are shown for each participant by group. Geometric mean is indicated by a horizontal line with whiskers representing 95% CI. *p*-values were calculated between groups within each timepoint using Wilcoxon rank-sum (Mann-Whitney U) test. Only significant *p*-values are shown. **(B)** Geometric Mean Fold Rise (GMFR) of MERS-CoV spike-binding IgG concentrations (IU/mL) from baseline are shown. Open symbols represent the GMFR for each group, whiskers represent the 95% CI. **(C)** Percent seroreactive participants in each group with four-fold or greater increase in MERS-CoV spike-binding IgG concentration (IU/mL) from baseline. Placebo groups are combined. MERS-CoV, Middle East respiratory syndrome coronavirus; MSD ECL, Meso Scale Discovery electrochemiluminescence; IgG, immunoglobulin G; IU, international units; mL, milliliters; CI, confidence interval; mg, milligram; Wks, weeks.

The MERS-CoV spike ECL assay revealed significant increases in IgG antibody levels above baseline for nearly all INO-4700 groups at Week 6 and all groups at Week 10 relative to Day 0, mirroring trends seen with RBD-binding responses (**Supplementary Table S3**). Likewise, upon comparing spike-binding antibody levels between groups at Week 6 and Week 10, the same trends emerged as were observed for RBD-binding antibodies (**Figure 4A**). However, only Group E showed a significant increase in MERS-CoV spike-binding IgG above baseline compared to placebo at both Week 6 and Week 10, while Group B and Group A had significant increases compared to placebo only at Week 6 and Week 10, respectively.

In all INO-4700 Groups, immunization led to an increase in the GMFR of MERS-CoV spike-specific IgG levels from baseline through Week 10 (**Figure 4B**), but these increases were lower than those observed for RBD-specific responses. At Week 10, Group E had the greatest GMFR (2.84), while the placebo group again showed little change (0.99). The rate of seroreactivity to MERS-CoV spike was similar to that observed for MERS-CoV RBD, with rates ranging from 19% for Group A to 32% for Group E at Week 10 (**Figure 4C**). The placebo group did have a low rate of seroreactivity to MERS-CoV spike based on *n* = 2 (6%) at Week 6 and *n* = 1 (3%) at Week 10. These findings confirm modest seroreactivity to spike protein across INO-4700 Groups, with minimal response seen in the placebo group.

**Figure 5:**
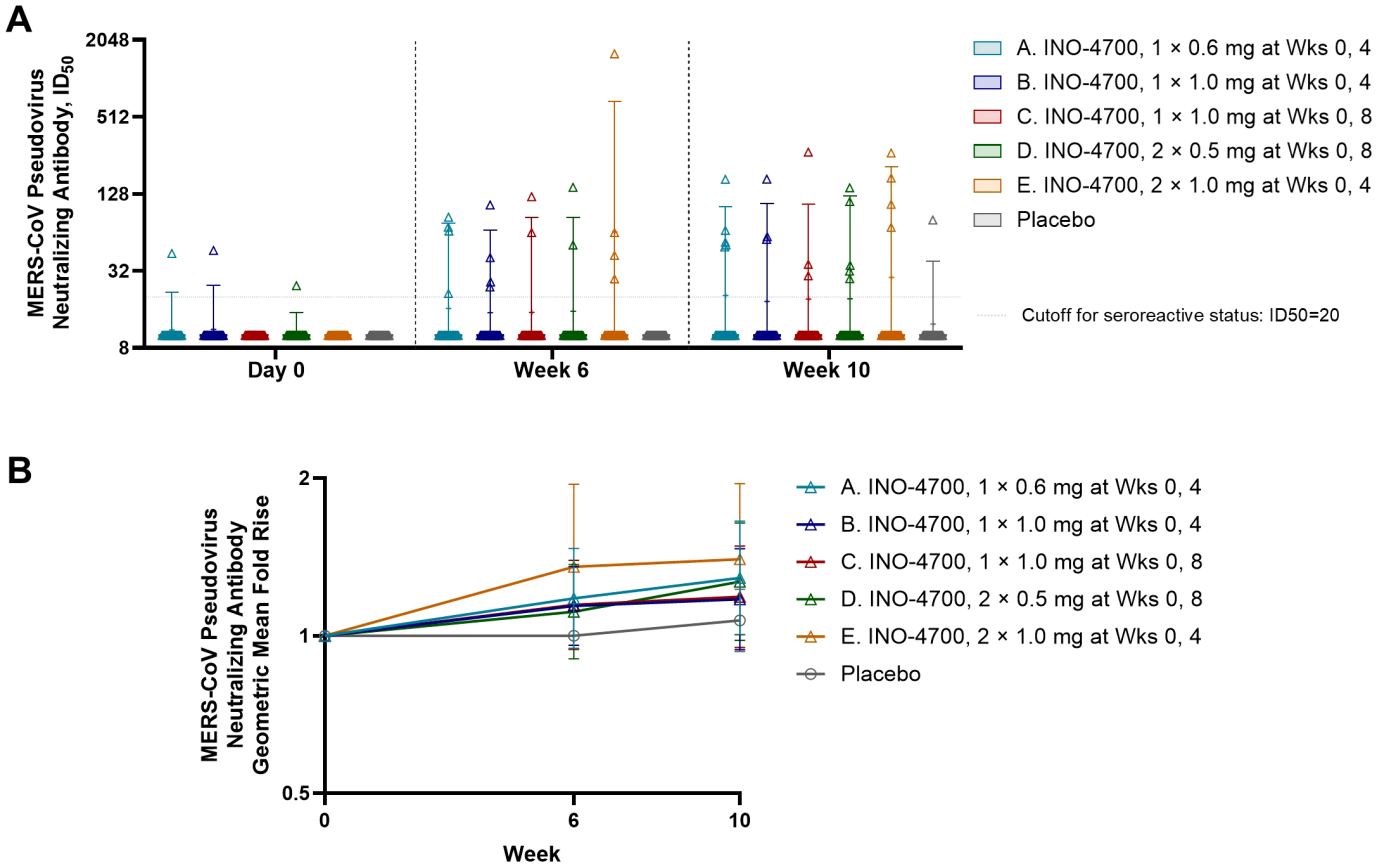
MERS-CoV spike-specific neutralizing antibody levels as measured by MERS spike-pseudotyped lentivirus analysis. **(A)** MERS-CoV pseudovirus neutralizing antibody levels (ID_50_) are shown for each participant by group. Placebo groups are combined. Box plots show the 25% and 75% percentiles with a horizontal line at the median and whiskers representing the 5% and 95% percentiles. Mean is represented by “+”. **(B)** The geometric mean fold rise (GMFR) of MERS-CoV pseudovirus neutralizing antibody levels (ID_50_) from baseline are shown for each group. Open symbols represent the GMFR; whiskers represent the 95% CI. MERS-CoV, Middle East respiratory syndrome coronavirus; ID_50_, 50% inhibitory dose; mg, milligram; Wks, weeks; CI, confidence interval.

Neutralizing antibody responses to INO-4700 were assessed using a MERS-CoV pseudovirus neutralizing assay, revealing limited response across all groups. Overall, antibody levels increased after immunization for very few participants from each of the INO-4700 groups, with no significant differences across timepoints within any group or between groups at any timepoint (**Figure 5A**). While the GMFR in MERS-CoV pseudovirus neutralizing antibodies increased for all INO-4700 groups over time as compared to placebo, the greatest GMFR observed at Week 10 was 1.40 in Group E. The lowest GMFR was 1.07 for the placebo group (**Figure 5B**). The rate of seroreactivity for MERS-CoV pseudovirus neutralizing antibodies in INO-4700 groups for any post-immunization timepoint ranged from 15% for Group C to 25% for Group A, as compared to 3% for the Placebo Group (**Table 5**).

**Table 5:** MERS-CoV seroreactivity based on pseudovirus neutralizing assays.

%^a^ (*n*/*N*)	Intervention groups (INO-4700)					Placebo^c^
	Group A: 1 × 0.6 mg at Wks 0, 4	Group B: 1 × 1.0 mg at Wks 0, 4	Group C: 1 × 1.0 mg at Wks 0, 8	Group D^b^: 2 × 0.5 mg at Wks 0, 8	Group E^b^: 2 × 1.0 mg at Wks 0, 4	
Week 6	13 (4/32)	13 (4/31)	6 (2/32)	6 (2/32)	13 (4/31)	0 (0/31)
Week 10	16 (5/32)	10 (3/30)	9 (3/33)	16 (5/32)	13 (4/31)	3 (1/31)
Any timepoint^d^	25 (8/32)	16 (5/31	15 (5/33)	22 (7/32)	19 (6/31)	3 (1/31)

MERS-CoV, Middle East respiratory syndrome coronavirus; %, percent; *n*, number of responders; *N*, total number of participants; mg, milligram; Wks,

weeks.

INO-4700 or placebo was administered intradermally (ID) into the deltoid area of the upper arms and was followed by electroporation (EP).

a. Rounded to the nearest whole number.

b. For Groups D and E receiving two doses of INO-4700 per visit, each dose was administered in the deltoid of different arms.

c. Placebo groups are combined.

d. Participants that had a response at either Weeks 6 or 10 as compared to baseline.

### Cellular immune response

**Figure 6:**
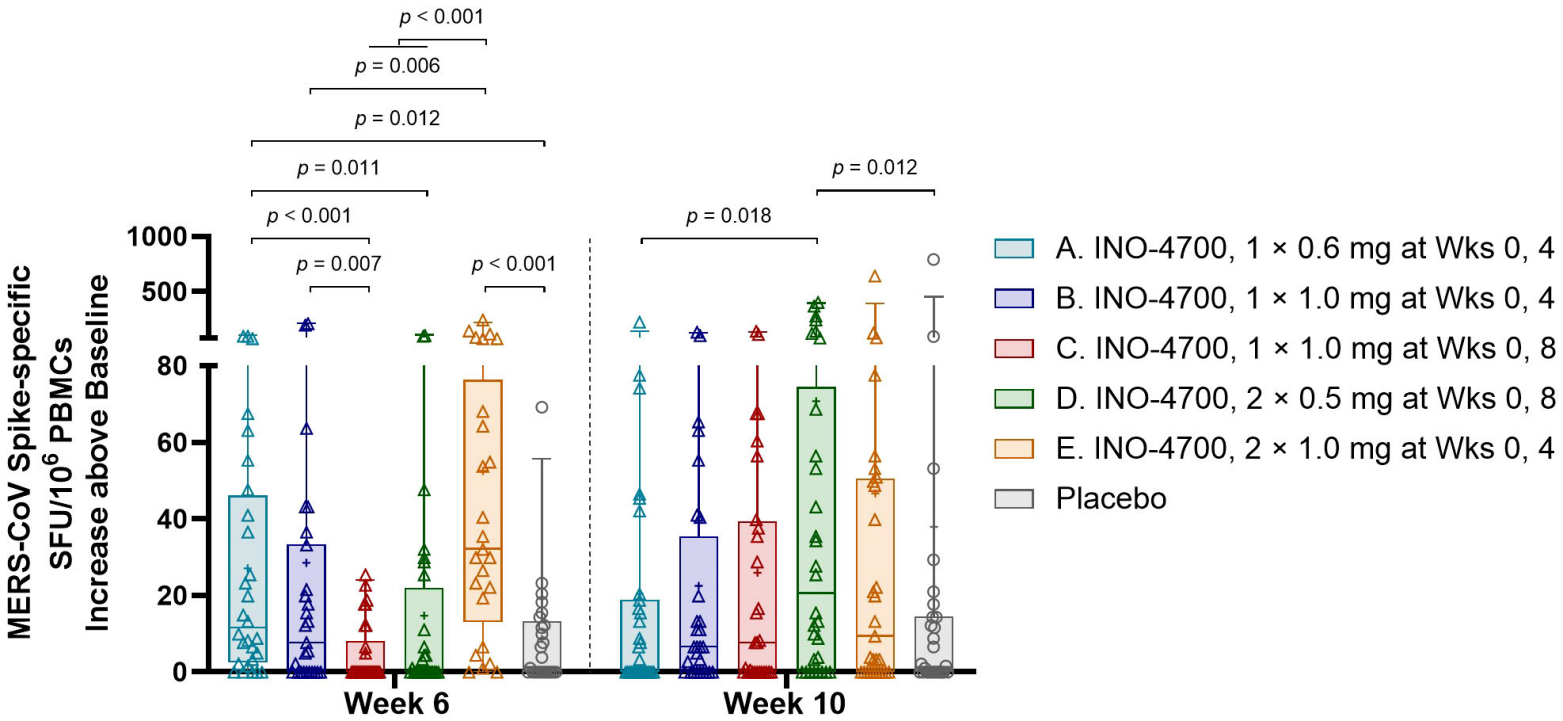
MERS-CoV spike-specific cellular immune responses as measured by IFN-γ ELISpot. The increase above baseline in MERS-CoV spike-specific SFU/10^6^ PBMCs, summing results for all 3 peptide pools, is shown for each participant by group. Placebo groups are combined. Box plots extend from 25th to 75th percentiles with a horizontal line at the median and whiskers extend from 5th to 95th percentiles. Mean is represented by “+”. *p-*values were calculated between groups within each timepoint using Wilcoxon rank-sum (Mann-Whitney U) test. Only significant *p-*values are shown. MERS-CoV, Middle East respiratory syndrome coronavirus; IFN-γ, interferon-gamma; ELISpot, enzyme-linked immunosorbent spot assay; SFU, spot forming unit; PBMCs, peripheral blood mononuclear cells; mg, milligram; Wks, weeks.

INO-4700 administration elicited a significant cellular immune response, as evidenced by elevated cytokine-secreting T cells in a MERS-CoV spike-specific interferon-gamma (IFN-γ) ELISpot assay. The sum of spot-forming units (SFUs) per 10^6^ PBMCs from stimulation with three separate, nonoverlapping peptide pools spanning the full sequence of MERS-CoV spike protein was used for analysis. A significant increase in SFUs per 10^6^ PBMCs above baseline was seen at both Weeks 6 and 10 for all INO-4700 and combined placebo groups (**Supplementary Table S4**). Furthermore, a significant increase was seen from Week 6 to Week 10 in Groups C and D, who received their second dose during Week 8. Comparison between INO-4700 groups and placebo showed significant increases over baseline in Groups A and E versus placebo at Week 6 and Group D versus placebo at Week 10 (**Figure 6**). Comparing INO-4700 groups at Week 6 revealed a significantly greater response in Group E versus Groups B, C, and D. Group A also showed a significantly greater increase in SFU per 10^6^ PBMCs above baseline relative to Groups C and D, both of which had received only one dose by Week 6. Interestingly, Group A did not show a statistical difference from Group B or Group E at Week 6, despite receiving the lowest dose on the same schedule. By Week 10, however, Group A showed the lowest increase in SFU per 10^6^ PBMCs above baseline of all INO-4700 groups and significantly less than Group D. The MERS-CoV spike-specific cellular immune response rate at any post-immunization timepoint ranged from 29% in Group C to 50% in Group E (**Table 6**). While the response rate rose from Week 6 to Week 10 for groups that received a second dose at Week 8, response rates declined from Week 6 to Week 10 for Groups A and E, who received their second dose at Week 4. The overall response rate for Placebo was 16%, which may have been due SARS-CoV-2 specific T-cells, derived from either SARS-CoV-2 infection or vaccination, having cross-reactive responses to MERS-CoV spike epitopes (31, 32).

**Table 6:** Cellular immune response to MERS-CoV spike protein as measured by IFN-γ ELISpot.

%^a^ (*n*/*N*)	Intervention groups (INO-4700)					Placebo^c^
	Group A: 1 × 0.6 mg at Wks 0, 4	Group B: 1 × 1.0 mg at Wks 0, 4	Group C: 1 × 1.0 mg at Wks 0, 8	Group D^b^: 2 × 0.5 mg at Wks 0, 8	Group E^b^: 2 × 1.0 mg at Wks 0, 4	
Week 6	39 (11/28)	22 (6/27)	7 (2/30)	21 (6/28)	48 (12/25)	12 (3/25)
Week 10	19 (6/31)	29 (8/28)	29 (8/28)	40 (12/30)	38 (11/29)	14 (4/29)
Any timepoint^d^	38 (12/32)	37 (11/30)	29 (9/31)	47 (15/32)	50 (15/30)	16 (5/31)

MERS-CoV, Middle East respiratory syndrome coronavirus; IFN-γ, interferon-gamma; ELISpot, enzyme-linked immunosorbent spot assay; %, percent;

*n*, number of responders; *N*, total number of participants; mg, milligram; Wks, weeks.

INO-4700 or placebo was administered intradermally (ID) into the deltoid area of the upper arms and was followed by electroporation (EP).

a. Rounded to the nearest whole number.

b. For Groups D and E receiving two doses of INO-4700 per visit, each dose was administered in the deltoid of different arms.

c. Placebo groups are combined.

d. Participants that had a response at either Weeks 6 or 10 as compared to baseline.

### SARS-CoV-2 cross-reactivity

Since the study was conducted during the SARS-CoV-2 pandemic, assays for binding antibodies to SARS-CoV-2 were used to assess the possibility of SARS-CoV-2 cross-reactivity and potential impact on INO-4700 seroconversion. More specifically, participant sera were evaluated for SARS-CoV-2 spike and NP-binding IgG. Most participants (~86%, 136/159) across all INO-4700 study groups were seroreactive (AU/mL > 1960) for SARS-CoV-2 spike at baseline (**Supplementary Figure S1A**). At Day 0, significant differences in SARS-CoV-2 spike-specific IgG concentrations among INO-4700 groups were noted. While these trends remained beyond Day 0, no significant differences in SARS-CoV-2 spike-specific IgG levels among INO-4700 groups were detected at Weeks 6 or 10. There were, however, significant differences between timepoints within all study groups, but INO-4700 immunization did not steadily increase or boost pre-existing SARS-CoV-2 spike binding antibodies over time (**Supplementary Table S5**). Increased SARS-CoV-2 IgG levels were observed for some participants at Weeks 6 or 10 compared to baseline, presumably due to SARS-CoV-2 infection that occurred post INO-4700 dosing. Conversely, some individuals had lower SARS-CoV-2 IgG levels at Weeks 6 or 10, possibly due to rapid waning of antibody levels typically observed for SARS-CoV-2 after infection or vaccination (33). Decreased or increased SARS-CoV-2 IgG levels for participants likely resulted in the significant differences observed within study groups.

The rate of participants seropositive for SARS-CoV-2 NP was lower than for SARS-CoV-2 spike – between 25 and 50% for all groups (AU/mL > 5000) (**Supplementary Figure S1B**). There were no significant differences in SARS-CoV-2 NP-specific IgG concentrations among groups, and only a few significant changes in concentrations within groups over time (**Supplementary Table S6**).

To investigate the potential impact of immune imprinting due to SARS-CoV-2 infection or vaccination on immune response to INO-4700, participants testing negative for both SARS-CoV-2 spike and NP by binding assay were evaluated across immunogenicity assessments (**Supplementary Table S7**). The humoral and cellular immune responses of SARS-CoV-2 seronegative participants administered INO-4700 were similar to those of all INO-4700 participants, the vast majority of which were SARS-CoV-2 seroreactive at any timepoint, which exceeded the immune responses observed in the placebo group by Week 10. These results suggest that SARS-CoV-2 antigen exposure did not interfere with the ability of INO-4700 to generate humoral and cellular immune responses.

To more precisely examine the potential impact of SARS-CoV-2 immune imprinting on the cellular immune response to INO-4700, responses to stimulation with each of three non-overlapping peptide pools covering the N-terminal (pool 1), middle (pool 2), or C-terminal (pool 3) regions of the MERS-CoV spike protein with varying sequence similarity to SARS-CoV-2 (48.2%, 52.0%, and 71.5%, respectively) were evaluated by IFN-γ ELISpot (**Supplementary Figure S2**). Cellular responses within each group at each timepoint were similar across all three peptide pools, regardless of percent sequence similarity to SARS-CoV-2, suggesting that exposure to SARS-CoV-2 infection or vaccination did not influence cellular response to INO-4700.

## Discussion

The threat posed by MERS-CoV, marked by a high case-fatality rate and lack of approved vaccine, underscores the critical need for safe and effective vaccine solutions (6). DNA vaccines, in particular, have great potential to fill this gap due to their thermostability at refrigerated temperatures, ability to be redosed, and suitability for rapid, large-scale manufacturing (10, 12).

In this Phase 2a clinical study (MERS-201), the DNA vaccine INO-4700 was found to be well-tolerated across all dose levels. TEAEs were primarily mild to moderate in severity, and comparable to placebo in frequency. Most TEAEs were Grades 1 and 2, six were Grade 3 and two Grade 4. Seventeen TEAEs were considered related to study treatment. These findings are consistent with earlier clinical trials (MERS-001 and MERS-002), where the same DNA vaccine (previously named GLS-5300), delivered intramuscularly (IM) and ID, was similarly well-tolerated in healthy volunteers with no vaccine-associated SAEs (12, 34). In MERS-001, the addition of a third dose did not alter the safety profile, further confirming the favorable safety profile observed to date for INO-4700 and this DNA vaccine platform, even with extended dosing regimens (12).

The highest immune responses in the current MERS-201 study were observed in Group E, which received the maximum cumulative INO-4700 dose (2 mg total at Weeks 0 and 4). This group exhibited significantly elevated RBD-binding and spike-binding IgG levels at Weeks 6 and 10 compared to lower dose groups. RBD-specific seroreactivity in Group E peaked at 42% by Week 10, while spike-specific seroreactivity reached 32%, which was the highest across all study arms. T cell responses, measured via ELISpot, further supported INO-4700 immunogenicity: spike-specific cellular immune response rates ranged from 29% in Group C to 50% in Group E.

In the MERS-001 study, IM-EP immunization of 0.67 to 6 mg of GLS-5300 vaccine resulted in <10% neutralizing responders after two immunizations (12). The current MERS-201 study also revealed limited induction of MERS pseudovirus neutralizing titers across the treated groups. The highest seroconversion rate of 42% was observed for RBD binding IgG for Group E at Week 10. In the MERS-001 study, seroconversion occurred in 59 (86%) of 69 participants and 61 (94%) of 65 participants after two and three vaccinations, respectively (12). The apparent reduced response rates seen in our study (MERS-201) are likely a consequence of relatively more stringent immune response criteria applied compared to the MERS-001 and MERS-002 trials. For MERS-201 as well as MERS-001 and MERS-002 binding assays, the response threshold was derived from the baseline assay signal for individual participants. In the previous trials, the response threshold was three standard deviations above baseline assay signal, while a higher threshold of four-fold baseline signal was used for MERS-201 (12).

The MERS-201 study showed that the INO-4700 MERS vaccine is safe and immunogenic in a 2-dose regimen. Though preclinical MERS challenge studies demonstrate that a two-dose regimen confers protection (25), clinical evidence also supports the benefit of a third dose of INO-4700 in augmenting the seroconversion rate and magnitude of humoral responses (MERS-001/002) (8, 11, 12, 35). Furthermore, such addition would still satisfy the WHO target product profile for a MERS-CoV vaccine dosing regimens of no more than 3 doses in prophylactic settings (36). Based on previous data, safety or tolerability concerns are not expected with a 3-dose regimen of the MERS vaccine (8, 12).

An important consideration in coronavirus prophylactic vaccine development is the potential for immune cross-reactivity, particularly with SARS-CoV-2, due to sequence homology with the respective spike protein sequences (37). In a previous study, Grobben et al. (35) reported SARS-CoV-2 mRNA immunization increases the levels of MERS-CoV spike and pre-existing endemic human CoV spike-binding IgGs. However, data from the present study suggests minimal impact of pre-existing SARS-CoV-2 immunity on INO-4700 seropositivity through immune imprinting. Despite approximately 86% of this study’s participants across all INO-4700 groups being SARS-CoV-2 spike seropositive at baseline, RBD or spike-binding IgG response rates following INO-4700 immunizations in participants previously exposed to SARS-CoV-2 S antigen (natural infection and/or vaccination) were not found to differ from rates in SARS-CoV-2 naïve, seronegative participants. INO-4700 immunization would be expected to trigger an anamnestic response leading to increased responses to cross-reactive antibody and T cell epitopes. However, INO-4700 immunization did not increase pre-existing antibodies that bind SARS-CoV-2 spike. Additionally, ELISpot responses did not support the occurrence of immune imprinting since after INO-4700 immunization the peptide pool with highest similarity to SARS-CoV-2 did not show any difference in the level of IFN-γ as compared to the peptide pools with less similarity. These findings indicate no evidence of immune imprinting occurring in the current MERS-201 study.

The development of safe and protective vaccines remains the goal for a prophylactic modality against MERS. Building upon preclinical and Phase 1 studies, the MERS-201 study demonstrated the safety, tolerability, and immunogenicity of INO-4700 administered ID and followed by EP as a two-dose regimen. Given the vaccine’s prospect as a preventative tool to mitigate an outbreak in endemic countries with large dromedary camel herds as a potential virus reservoir, further evaluation would be warranted to improve upon the humoral responses demonstrated in this study as it was initially illustrated in the MERS-001 (12) and MERS-002 (34) clinical studies. Continued development of this DNA-based vaccine would ensure preparedness for future MERS-CoV outbreaks after initial preventive vaccination campaigns, particularly given its potential for redosing to enhance long-term protection. By avoiding anti-vector immunity, DNA vaccines offer a distinct advantage over viral-based vector platforms, positioning them as a promising tool for repeatable immunization strategies that have the potential to offer durable protection (12, 14, 15).

## Data Availability

The datasets presented in this article are not readily available because restrictions may apply due to privacy reasons, regulatory submissions, and/or ongoing research projects. Requests to access the datasets should be directed to Laurent Humeau, Laurent.Humeau@inovio.com.
