# Supplementary material for: Safety, tolerability, and immunogenicity of a DNA-based vaccine (INO-4700) against Middle East respiratory syndrome coronavirus: phase 2a study in healthy volunteers
Source: Front Immunol. 2025 Nov 14;16:1662923. doi: 10.3389/fimmu.2025.1662923 (PMC12660258; doi:10.3389/fimmu.2025.1662923)
Supplement: Supplementary file 1 [file DataSheet1.pdf]

Supplementary Table 1. All Grade ≥ 3 and Serious Treatment-Emergent Adverse Events in the Safety Population of MERS-201 Study

	Intervention Groups (INO-4700)						
	Group A (*N*=32)	Group B (*N*=33)	Group C (*N*=32)	Group D[a] (*N*=32)	Group E[a] (*N*=31)	Total Intervention (*N*=160)	All Groups Combined (*N*=192)
	1 × 0.6 mg at Wks 0, 4	1 × 1.0 mg at Wks 0, 4	1 × 1.0 mg at Wks 0, 8	2 × 0.5 mg at Wks 0, 8	2 × 1.0 mg at Wks 0, 4		
Type of Event/Preferred Term	*n* (%)	*n* (%)	*n* (%)	*n* (%)	*n* (%)	*n* (%)	*n* (%)
**Total Number of Grade ≥ 3 TEAEs**	**1**	**1**	**5**	**0**	**1**	**8**	**8**
**Number of Participants with Grade ≥ 3 TEAEs**	**1 (3.1)**	**1 (3.0)**	**3 (9.4)**	**0 (0.0)**	**1 (3.2)**	**6 (3.8)**	**6 (3.1)**
Alanine aminotransferase increased	0 (0.0)	0 (0.0)	2 (6.3)	0 (0.0)	0 (0.0)	2 (1.3)	2 (1.0)
Aspartate aminotransferase increased	0 (0.0)	0 (0.0)	2 (6.3)	0 (0.0)	0 (0.0)	2 (1.3)	2 (1.0)
Hypernatremia	1 (3.1)	0 (0.0)	0 (0.0)	0 (0.0)	1 (3.2)	2 (1.3)	2 (1.0)
Hypoglycemia[b]	0 (0.0)	0 (0.0)	1 (3.1)	0 (0.0)	0 (0.0)	1 (0.6)	1 (0.5)
Soft tissue injury[b]	0 (0.0)	1 (3.0)	0 (0.0)	0 (0.0)	0 (0.0)	1 (0.6)	1 (0.5)
**Total Number of Serious Adverse Events (SAEs)**	**0**	**1**	**0**	**1**	**0**	**2**	**2**
**Number of Participants with ≥ 1 SAE**	**0 (0.0)**	**1 (3.0)**	**0 (0.0)**	**1 (3.1)**	**0 (0.0)**	**2 (1.3)**	**2 (1.0)**
Soft tissue injury	0 (0.0)	1 (3.0)	0 (0.0)	0 (0.0)	0 (0.0)	1 (0.6)	1 (0.5)
Tendon injury	0 (0.0)	0 (0.0)	0 (0.0)	1 (3.1)	0 (0.0)	1 (0.6)	1 (0.5)

N/n, number of participants; mg, milligram; Wks, weeks; TEAE, treatment-emergent adverse event; SAE, serious adverse event.
A TEAE was defined as any AE that occurs within 30 days of the last treatment.
INO-4700 or placebo was administered intradermally (ID) into the deltoid area of the upper arms and was followed by electroporation (EP).
a. For Groups D and E receiving two doses of INO-4700 per visit, each dose was administered in the deltoid of different arms.
b. Grade 4 events.
