# Supplementary material for: Safety, tolerability, and immunogenicity of a DNA-based vaccine (INO-4700) against Middle East respiratory syndrome coronavirus: phase 2a study in healthy volunteers
Source: Front Immunol. 2025 Nov 14;16:1662923. doi: 10.3389/fimmu.2025.1662923 (PMC12660258; doi:10.3389/fimmu.2025.1662923)
Supplement: Supplementary file 2 [file DataSheet2.pdf]

Supplementary Table 2. Statistical Comparison of MERS-CoV RBD-binding IgG Concentrations (IU/ml) Above Baseline

	Exact *p*-value[a]		
**Study Group**	**Wk 6 vs D0**	**Wk 10 vs D0**	**Wk 10 vs Wk 6**
**A: 1 × 0.6 mg at Wks 0, 4**	<0.001	<0.001	0.495
**B: 1 × 1.0 mg at Wks 0, 4**	<0.001	<0.001	0.054
**C: 1 × 1.0 mg at Wks 0, 8**	0.016	0.004	0.009
**D[b]: 2 × 0.5 mg at Wks 0, 8**	0.002	<0.001	0.002
**E[b]: 2 × 1.0 mg at Wks 0, 4**	<0.001	<0.001	0.105
**Placebo[c]**	0.250	0.250	0.813

MERS-CoV, Middle East respiratory syndrome coronavirus; RBD, receptor binding domain; IgG, immunoglobulin G; IU, international units; ml, milliliters; Wk(s), week(s); D0, day 0; mg, milligram.
INO-4700 or placebo was administered intradermally (ID) into the deltoid area of the upper arms and was followed by electroporation (EP).
a. *p*-values were calculated between timepoints within each group using the Wilcoxon signed-rank test.
b. For Groups D and E receiving two doses of INO-4700 per visit, each dose was administered in the deltoid of different arms.
c. Placebo groups are combined.
