# Supplementary material for: Safety, tolerability, and immunogenicity of a DNA-based vaccine (INO-4700) against Middle East respiratory syndrome coronavirus: phase 2a study in healthy volunteers
Source: Front Immunol. 2025 Nov 14;16:1662923. doi: 10.3389/fimmu.2025.1662923 (PMC12660258; doi:10.3389/fimmu.2025.1662923)
Supplement: Supplementary file 5 [file DataSheet5.pdf]

Supplementary Table 5. Statistical Comparison of SARS-CoV-2 Spike-binding IgG Concentrations between Timepoints within each Study Group

**Study Group**	**Exact *p*-value**[a]		
	Wk 6 vs D0	Wk 10 vs D0	Wk 10 vs Wk 6
**A: INO-4700, 1 × 0.6 mg at Wks 0, 4**	0.006	0.022	0.004
**B: INO-4700, 1 × 1.0 mg at Wks 0, 4**	0.636	0.824	0.046
**C: INO-4700, 1 × 1.0 mg at Wks 0, 8**	0.027	0.034	<0.001
**D[b]: INO-4700, 2 × 0.5 mg at Wks 0, 8**	0.003	0.043	0.004
**E[b]: INO-4700, 2 × 1.0 mg at Wks 0, 4**	0.003	0.030	0.002
**Placebo[c]**	0.079	0.480	0.015

SARS-CoV-2, severe acute respiratory syndrome coronavirus 2; IgG, immunoglobulin G; Wk(s), week(s); D0, day 0; mg, milligram.

INO-4700 or placebo was administered intradermally (ID) into the deltoid area of the upper arms and was followed by electroporation (EP).

a. *p*-values were calculated between timepoints within each group using the Wilcoxon signed-rank test.

b. For Groups D and E receiving two doses of INO-4700 per visit, each dose was administered in the deltoid of different arms.

c. Placebo groups are combined.
