# Supplementary material for: Safety, tolerability, and immunogenicity of a DNA-based vaccine (INO-4700) against Middle East respiratory syndrome coronavirus: phase 2a study in healthy volunteers
Source: Front Immunol. 2025 Nov 14;16:1662923. doi: 10.3389/fimmu.2025.1662923 (PMC12660258; doi:10.3389/fimmu.2025.1662923)
Supplement: Supplementary file 6 [file DataSheet6.pdf]

Supplementary Table 6. Statistical Comparison of SARS-CoV-2 NP-binding IgG Concentrations between Timepoints within each Study Group

**Study Group**	**Exact *p*-value[a]**		
	Wk 6 vs D0	Wk 10 vs D0	Wk 10 vs Wk 6
**A: INO-4700, 1 × 0.6 mg at Wks 0, 4**	0.012	0.524	0.185
**B: INO-4700, 1 × 1.0 mg at Wks 0, 4**	0.961	0.367	0.039
**C: INO-4700, 1 × 1.0 mg at Wks 0, 8**	0.296	0.447	0.367
**D[b]: INO-4700, 2 × 0.5 mg at Wks 0, 8**	0.015	0.304	0.014
**E[b]: INO-4700, 2 × 1.0 mg at Wks 0, 4**	0.066	0.399	0.204
**Placebo[c]**	0.041	0.410	0.120

SARS-CoV-2, severe acute respiratory syndrome coronavirus 2; NP, nucleocapsid protein; IgG, immunoglobulin G; Wk(s), week(s); D0, day 0; mg, milligram.
INO-4700 or placebo was administered intradermally (ID) into the deltoid area of the upper arms and was followed by electroporation (EP).
a. *p*-values were calculated between timepoints within each group using the Wilcoxon signed-rank test.
b. For Groups D and E receiving two doses of INO-4700 per visit, each dose was administered in the deltoid of different arms.
c. Placebo groups are combined.
