# Supplementary material for: Safety, tolerability, and immunogenicity of a DNA-based vaccine (INO-4700) against Middle East respiratory syndrome coronavirus: phase 2a study in healthy volunteers
Source: Front Immunol. 2025 Nov 14;16:1662923. doi: 10.3389/fimmu.2025.1662923 (PMC12660258; doi:10.3389/fimmu.2025.1662923)
Supplement: Supplementary file 7 [file DataSheet7.pdf]

Supplementary Table 7. MERS-CoV-specific Readouts for Humoral and Cellular Immune Responses from Participants Negative for SARS-CoV-2 at All Timepoints

	RBD-binding IgG above Baseline (IU/ml)		Spike-binding IgG above Baseline (IU/ml)		Pseudovirus neutralizing antibody (ID_{50})		Spike-specific IFN-γ SFU per 10^6 PBMCs above Baseline	
**Week 6**	**INO-4700[a]**	**Placebo[b]**	**INO-4700[a]**	**Placebo[b]**	**INO-4700[a]**	**Placebo[b]**	**INO-4700[a]**	**Placebo[b]**
n	10	4	10	4	10	4	8	4
Mean[c]	1.5	1.0	2.1	1.0	10.0	10.0	6.4	7.5
Median	1.0	1.0	1.3	1.0	10.0	10.0	3.9	7.2
95% CI	-2.4, 10.3	1.0, 1.0	0.1, 7.7	1.0, 1.0	10.0, 10.0	10.0, 10.0	0.0, 12.8	-6.3, 21.3
% Response	0.00%	0.00%	30.0%	0.0%	0.0 %	0.0 %	12.5%	25.0%
**Week 10**								
n	10	4	10	4	10	4	9	4
Mean[c]	1.8	1.0	2.4	1.0	25.6	10.0	10.6	8.6
Median	1.0	1.0	1.0	1.0	10.0	10.0	0.0	8.3
95% CI	-1.7, 11.4	1.0, 1.0	-0.4, 11.8	1.0, 1.0	-9.7, 61.0	10.0, 10.0	-5.8, 27.0	-5.4, 22.6
% Response	10.0%	0.0%	30.0%	0.0%	10.0 %	0.0 %	11.1%	0.0%

MERS-CoV, Middle East respiratory syndrome coronavirus; SARS-CoV-2, severe acute respiratory syndrome coronavirus 2; RBD, receptor binding domain; IgG, immunoglobulin G; IU, international units; ml, milliliters; ID_{50}, 50% inhibitory dose; IFN-γ, interferon-gamma; SFU, spot forming unit; PBMCs, peripheral blood mononuclear cells; n, number of participants; CI, confidence interval.

INO-4700 or placebo was administered intradermally (ID) into the deltoid area of the upper arms and was followed by electroporation (EP).

a. INO-4700 groups are combined.

b. Placebo groups are combined.

c. Geometric mean for RBD- and Spike-binding IgG.
