# Supplementary figures and images for: Safety, tolerability, and immunogenicity of a DNA-based vaccine (INO-4700) against Middle East respiratory syndrome coronavirus: phase 2a study in healthy volunteers
Source: Front Immunol. 2025 Nov 14;16:1662923. doi: 10.3389/fimmu.2025.1662923 (PMC12660258; doi:10.3389/fimmu.2025.1662923)

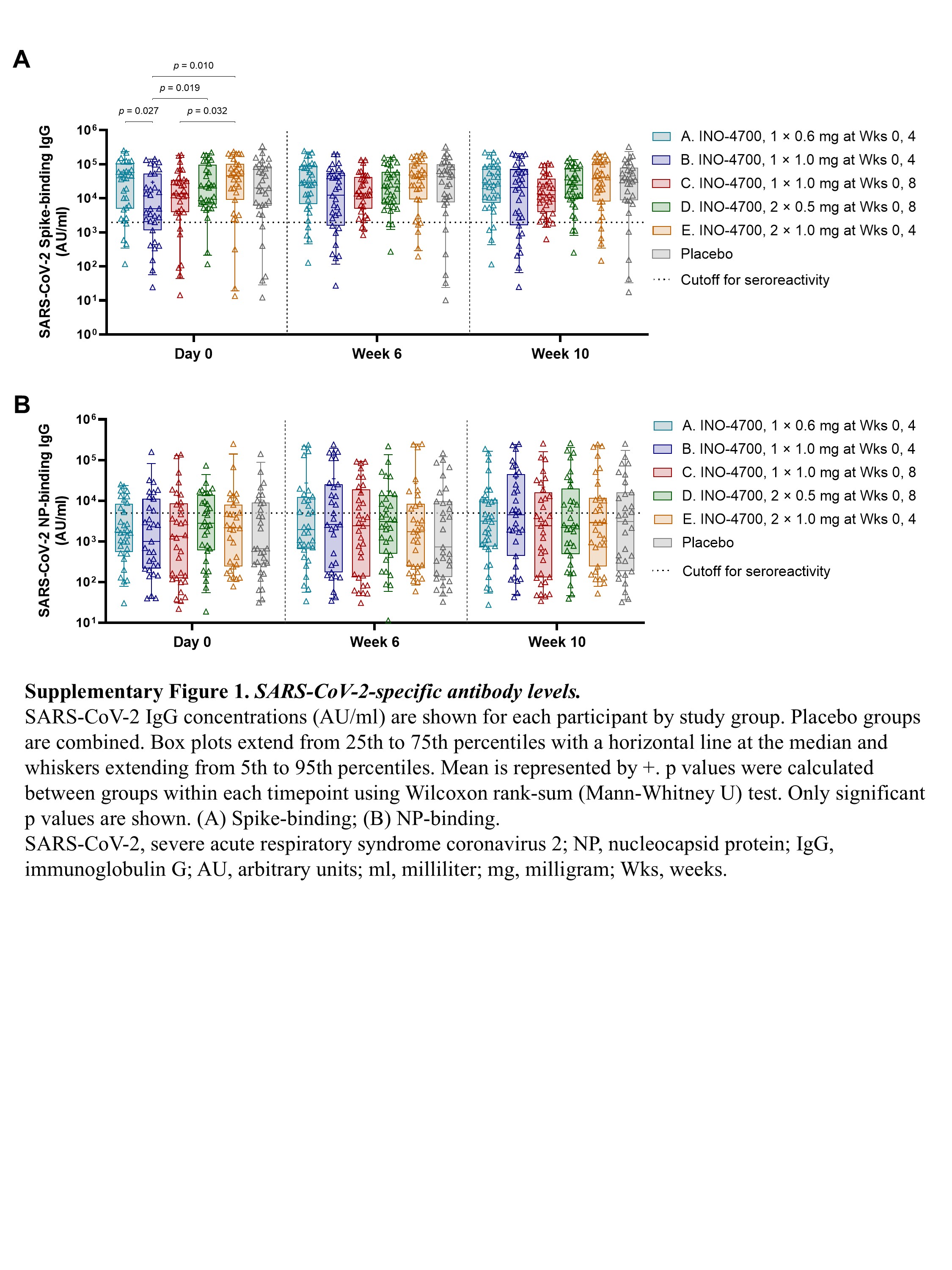

Supplement: Supplementary file 8 [file Image1.jpeg]

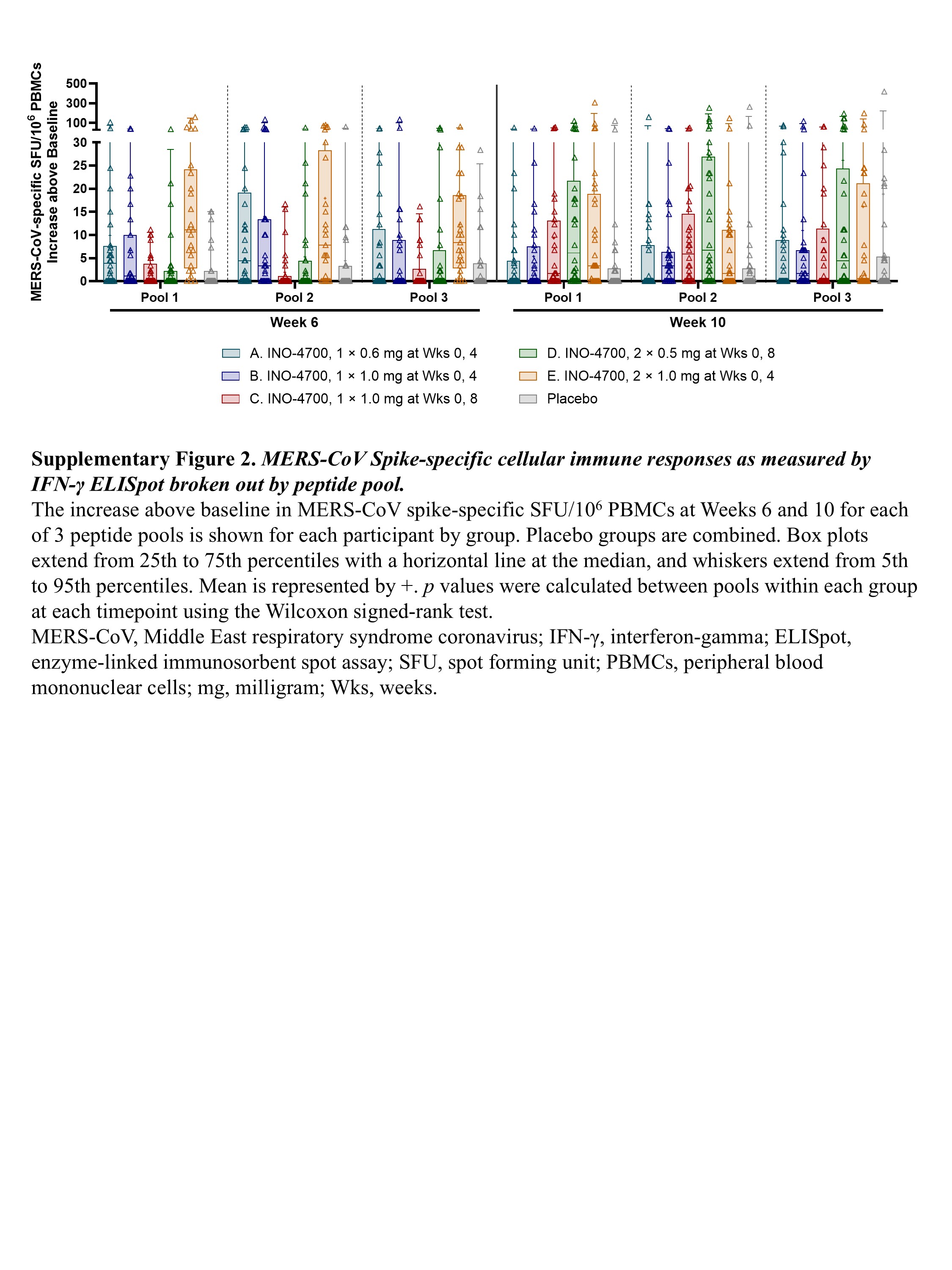

Supplement: Supplementary file 9 [file Image2.jpeg]
